# Unusual Anti-allergic Diterpenoids from the Marine Sponge *Hippospongia lachne*

**DOI:** 10.1038/srep43138

**Published:** 2017-02-22

**Authors:** Li-Li Hong, Hao-Bing Yu, Jie Wang, Wei-Hua Jiao, Bao-Hui Cheng, Fan Yang, Yong-Jun Zhou, Bin-Bin Gu, Shao-Jiang Song, Hou-Wen Lin

**Affiliations:** 1 Research Center for Marine Drugs, State Key Laboratory of Oncogenes and Related Genes, Department of Pharmacy, Renji Hospital, School of Medicine, Shanghai Jiao Tong University, Shanghai 200127, P. R. China; 2 Department of Natural Products Chemistry, Shenyang Pharmaceutical University, Shenyang 110016, P. R. China; 3 Shenzhen Key Laboratory of ENT, Longgang ENT hospital & Institute of ENT, Shenzhen 518172, P. R. China

## Abstract

Hipposponlachnins A (**1**) and B (**2**), possessing an unprecedented tetracyclo [9.3.0.0^2,8^.0^3,7^] tetradecane ring system, and the probable biogenetic precursor [**3**, (1R*,2*E*,4R*,7*E*,10S*,11S*,12R*)-10, 18-diacetoxydolabella-2,7-dien-6-one] of **1**‒**2** were isolated from the South China Sea marine sponge *Hippospongia lachne*. The structures of the novel compounds were determined using integrated spectroscopic methods in combination with single-crystal X-ray diffraction analysis. Compounds **1**‒**2** showed potent inhibitory activity on the release of *β*-hexosaminidase, a biomarker for degranulation, as well as the production of pro-inflammatory cytokine IL-4 and lipid mediator LTB_4_ in DNP-IgE-stimulated RBL-2H3 cells.

Allergy is an abnormal adaptive immune response against allergens, such as pollen, food, dust mites, cosmetics, mold spores, and animal hairs^1^,^2^. *β*-Hexosaminidase, typically released in addition to histamine by mast cells after stimulation with antigen, has been used as a biomarker for antigen-induced degranulation in rat basophilic leukemia (RBL-2H3) cells^3^. Thus, the release of *β*-hexosaminidase in RBL-2H3 cells has commonly been used as a reliable indicator of inhibitory activity against allergic reactions induced by natural products^4^. Natural products have been proved to be an important source for drug discovery and development. More than three hundreds natural compounds were reported to exhibit diverse biological properties, such as antitumor, antibacterial, antifungal, and a range of other bioactivities^5^. However, only a few natural compounds with anti-allergic activity have been isolated to date^5^. Therefore, there is a great opportunity in search for leads to treat allergy effectively.

Marine invertebrates such as sponges are well-known sources of bioactive natural products^6^. Sponges of the genus *Hippospongia* are reported to be rich sources of polyketides^7^, sesquiterpenes^8^, sesterterpenes and sulfates^9^, furanoterpenes^10^, triterpenoic acids^11^, fatty acids^12^, and sterols^13^. These metabolites exhibited a broad spectrum of biological activities, including cytotoxic^9^, antifungal^7^, anti-inflammatory^8^, coronary vasodilating^14^, and RCE-protease inhibitory activities^11^. A literature survey revealed that no anti-allergic metabolites have been isolated from sponges belonging to this genus. As part of our searching for anti-allergic secondary metabolites from marine sponges, we investigated the sponge *Hippospongia lachne*, collected from the Xisha Islands of China in March 2013. The 95% ethanol extract of *H. lachne* showed promising *β*-hexosaminidase inhibitory activity with IC_50_ value of 7.8 μg/mL. Further bioactivity-guided fractionation of this extract led to the isolation of compounds **1**‒**3** (Fig. 1). Of these three metabolites, hipposponlachnins A (**1**) and B (**2**) possessed a rare tetracyclo [9.3.0.0^2,8^.0^3,7^] tetradecane carbon skeleton, exemplifying the first diterpene scaffold obtained from marine source, and showed inhibitory activity on the release of *β*-hexosaminidase in anti-murine DNP-IgE-stimulated RBL-2H3 cells. Herein, we report the isolation, structure elucidation and anti-allergic activity of the two unprecedented diterpenoids. In addition, biogenetic relationship between these compounds was proposed, suggesting **1**‒**2** may be obtained from **3** via an intramolecular [2 + 2] cycloaddition reaction.

## Results and Discussion

### Isolation and structure elucidation

**Figure 1:**
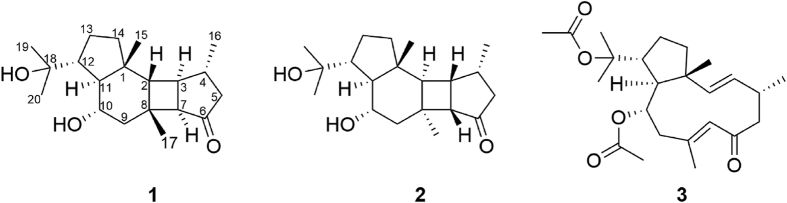
Structures of compounds 1‒3.

Air dried specimen of *H. lachne* were extracted with 95% ethanol and after evaporation under reduced pressure, the aqueous residue was extracted with EtOAc. The EtOAc layer was repeatedly chromatographed on Sephadex LH-20, silica gel, ODS column and reversed phase high performance liquid chromatography (HPLC) to yield hipposponlachnin A (**1**), hipposponlachnin B (**2**), and (1R*,2*E*,4R*,7*E*,10S*,11S*,12R*)-10,18-diacetoxydolabella-2,7-dien-6-one (**3**) (Fig. 1).

Hipposponlachnin A (**1**) was obtained as a colorless crystal. Its molecular formula was determined to be C_20_H_32_O_3_ by HRESIMS (*m/z* 343.2251 [M + Na]^+^), indicating five degrees of unsaturation. The IR spectrum featured characteristic absorptions of hydroxyl (3280 cm^−1^) and carbonyl (1732 cm^−1^) groups. The ^1^H NMR spectrum revealed the presence of four methyl singlets (*δ*_H_ 0.85, 1.11, 1.24, and 1.28), one methyl doublet (*δ*_H_ 0.97, d, *J* = 7.0 Hz), and one oxymethine proton (*δ*_H_ 3.72, td, *J* = 10.0, 4.5 Hz). The ^13^C and DEPT135 NMR spectra displayed 20 carbon resonances (Table 1), including five methyls, four methylenes, seven methines (one oxygenated), and four quaternary carbons (one oxygenated and one carbonyl). The aforementioned data accounted for one degree of unsaturation. The remaining four degrees of unsaturation suggested a tetracyclic core in **1**.

**Figure 2:**
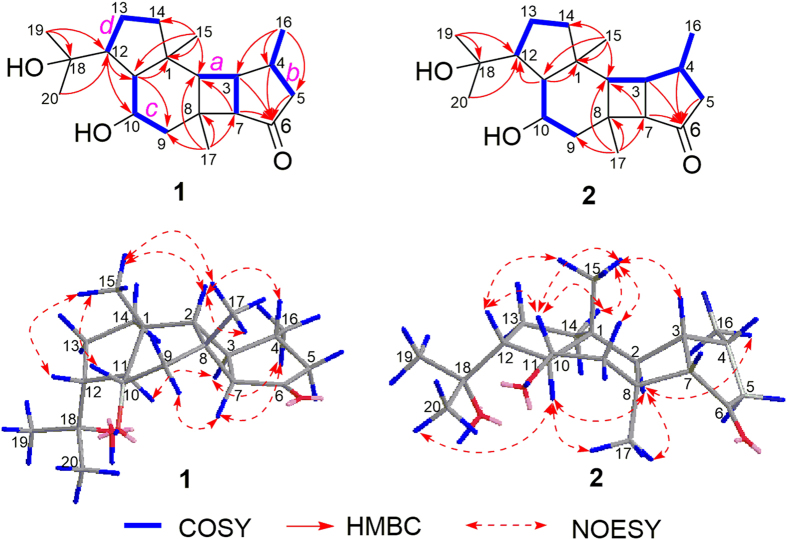
Key HMBC and COSY and selected NOE correlations of 1 and 2.

The planar structure of **1** was elucidated via a detailed analysis of 2D NMR data (Fig. 2). The COSY spectrum readily revealed the presence of four isolated spin systems: (*a*) C-2‒C-3‒C-7, (*b*) C-5‒C-4‒C-16, (*c*) C-9‒C-10‒C-11, and (*d*) C-12‒C-13‒C-14. The observed HMBC correlations from H_3_-16 to C-3, C-4, and C-5 and from H-12 to C-11 revealed the connectivities of C-3 and C-4 and of C11 and C-12, respectively. The HMBC correlations from H-3, H-4, H-5, and H-7 to C-6 indicated that C-5 and C-7 were connected via the carbonyl carbon C-6. The additional HMBC correlations of H_3_-17/C-2, C-7, C-8, and C-9, H_3_-15/C-1, C-2, C-11, and C-14, and H-12/C-10, tethered the remaining three fragments *a, c*, and *d* by inserting the “loose ends” of the quaternary carbons C-1 and C-8, and located the three methyl groups at C-1, C-4, and C-8, respectively, demonstrating a tetracyclo [9.3.0.0^2,8^.0^3,7^] tetradecane core in **1**. Moreover, the presence of a hydroxy isopropyl group at C-12 was supported by the HMBC correlations from the two remaining methyl groups, H_3_-19 and H_3_-20, to C-12 and the oxygenated quaternary carbon at *δ*_C_ 72.7 (C-18). The unassigned hydroxyl group was attached at the downfield-shifted carbon at (*δ*_C_ 67.2, C-10). Thus, the planar structure of hipposponlachnin A (**1**) was established as shown in Fig. 2.

**Figure 3:**
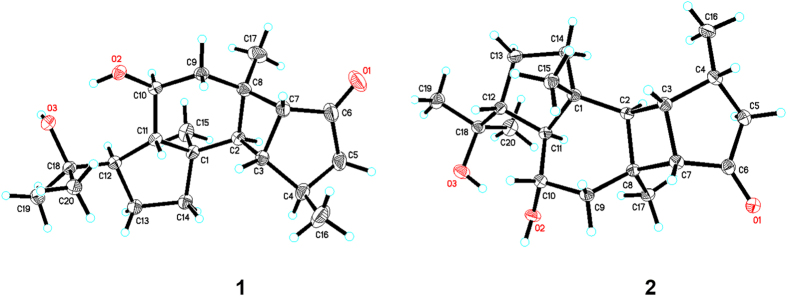
X-ray crystallographic structures of 1 and 2.

The relative configuration of **1** was determined by NOESY data (Fig. 2). The NOESY correlations of H_3_-15/H-2, H_3_-15/H_3_-17, H_3_-15/H-10, H_3_-15/H-12, H-2/H-4, and H-2/H_3_-17 indicated that these protons and methyl groups were cofacially oriented, whereas the NOESY correlations of H-11/H-3, H-3/H_3_-16, H_3_-16/H-7, and H-7/H-9*α* suggested that they were oriented in opposite directions. Therefore, the configuration of **1** was determined and confirmed by single-crystal X-ray diffraction analysis with Cu K*α* irradiation as shown in Fig. 3.

Hipposponlachnin B (**2**) was also obtained as a colorless crystal. Its molecular formula of C_20_H_32_O_3_ was deduced from its HRESIMS data, consistent with that of **1**. The overall appearance of the NMR spectrum of **2** revealed close structural similarity between **1** and **2**, indicating the presence of the same tetracyclo [9.3.0.0^2,8^.0^3,7^] tetradecane skeleton in both compounds. Further analysis of 1D and 2D NMR spectra of **2** established the planar structure of **2**, which was identical to that of **1** (Fig. 2). Moreover, the almost mirror-image CD spectra of **1** and **2** (see Supplementary Fig. S21) indicated that these two compounds must be a pair of diastereoisomers with a variation of configurations at the chiral centers around the carbonyl chromophore. Detailed analysis of the single-crystal X-ray diffraction patterns of **2** confirmed this hypothesis and established the absolute configuration of **2** as 1*R*,2*R*,3*S*,4*R*,7*S*,8*S*,10*S*,11*S*,12*R* (Fig. 3).

Compound **3** was identified as a known dolabellane diterpene, named (1R*,2*E*,4R*,7*E*,10S*,11S*,12R*)-10,18-diacetoxydolabella-2,7-dien-6-one, by comparing its physical and spectroscopic features with the data reported in the literature^15^,^16^.

**Figure 4:**
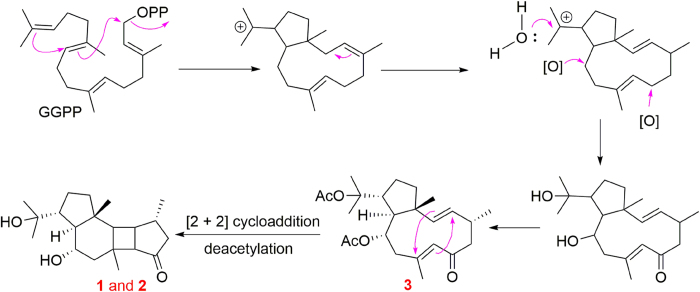
Proposed Biosynthetic Pathway of 1–3.

In addition, the biosynthetic pathway for **1**‒**3** was proposed as shown in Fig. 4. **3** could be produced from geranylgeranyl diphosphate (GGPP) following a series of biosynthetic cyclization, migration, oxidation, and acetylation processes^17^,^18^,^19^. The four-membered ring in **1** and **2** might be formed from **3** via an intramolecular [2 + 2] cycloaddition reaction between two double bonds (Δ^2,3^ and Δ^7,8^)^7^,^20^. The deacetylation could be carried out by an esterase^21^. What’s more, compound **3** with 7, 8-E geometry rather than 7,8-Z isomer is the predicted precursor of **1** and **2**, because the high rotational barrier around the C_7_-C_8_ bond in the intermediate of the 7, 8-Z isomer in the [2 + 2] cycloaddition process makes it difficult to form stable *trans*-cyclobutane products (**1** and **2**) (see Supplementary Fig. S26).

### Anti-allergic activity evaluation

**Figure 5:**
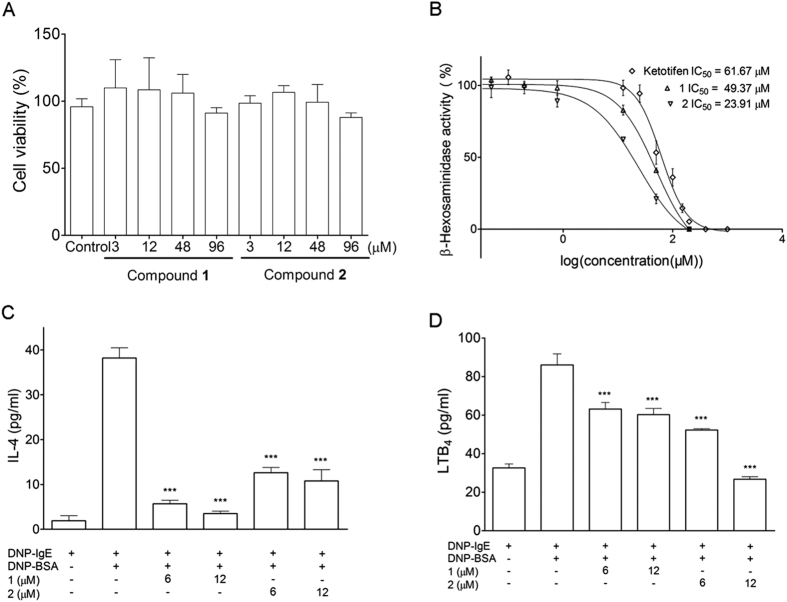
(**A**) Cytotoxic effects of **1** and **2** on RBL-2H3 cells. (**B**) Inhibitory effects of **1** and **2** on the release of *β*-hexosaminidase in DNP-IgE-activated RBL-2H3 cells. (**C**) Inhibition of IL-4 production of **1** and **2** in RBL-2H3 cells. (**D**) Inhibitory activity on lipid mediator LTB_4_ secretion of **1** and **2** in RBL-2H3 cells. Data are presented as the mean ± SD values of triplicate determinations. *P < 0.05, **P < 0.01, ***P < 0.001 versus DNP-BSA-treated group (n = 3). Data are analyzed by one-way ANOVA followed by Tukey’s Multiple Comparison Post-Test (GraphPad Prism 5.0).

Subsequently, RBL-2H3 cells were used as a model system to evaluate the anti-allergic activity of **1**‒**2**^22^. As indicated in Fig. 5A, no significant cytotoxicity was observed in RBL-2H3 cells after 24 h of treatment with **1** or **2**. **1** and **2** exhibited higher activity (IC_50_ 49.37 and 23.91 *μ*M, respectively) in the release of *β*-hexosaminidase inhibition (Fig. 5B), compared with the market-available anti-asthmatic drug, ketotifen fumarate (IC_50_ = 63.88 *μ*M). In addition, **1** and **2** suppressed IL-4 production in a dose-dependent manner (Fig. 5C) and significantly inhibited LTB4 release in activated RBL-2H3 cells compared with untreated control (Fig. 5D). The results indicated that **1** and **2** are promising new anti-allergic lead compounds.

In summary, two novel tetracyclic diterpenes (**1**‒**2**) were isolated from the marine sponge *H. lachne*, together with their probable biogenetic precursor (**3**). The structures of new compounds were elucidated by spectroscopic and single-crystal X-ray diffraction analysis. To our knowledge, only one diterpenoid with a similar skeleton, namely vulgarisin A, derived from the Chinese Medicinal Plant *Prunella vulgaris*, has been reported to date^19^. These two novel compounds represented an unprecedented tetracyclo [9.3.0.0^2,8^.0^3,7^] tetradecane ring system from marine source for the first time. Moreover, the potent inhibitory activity on the release of *β*-hexosaminidase for **1**‒**2** suggested that these two bioactive diterpenoids can be the potential therapeutic agents for the treatment of allergy.

## Methods

### General Experimental Procedures

Optical rotation data were recorded on a PerkinElmer model 341 polarimeter with a 10 cm length cell at room temperature. UV and IR (KBr) spectra were obtained on a Hitachi U-3010 spectrophotometer and Jasco FTIR-400 spectrometer, respectively. CD spectra were obtained on a Jasco J-715 spectropolarimeter in MeCN. NMR spectra including 1D and 2D spectra were acquired at room temperature on Bruker AMX-500 instrument. HRESIMS data were obtained on a Waters Q-Tof micro YA019 mass spectrometer. Reversed-phase HPLC was performed on YMC-Pack Pro C18 RS (5 *μ*m) columns with a Waters 1525 separation module equipped with a Waters 2998 photodiode array detector. Purifications by column chromatography were performed on silica gel 60 (200–300 mesh; Yantai, China), Sephadex LH-20 (18–110 *μ*m, Pharmacia Co.), and ODS (50 *μ*m, YMC Co.). Analytical thin-layer chromatography was carried out using HSGF 254 plates and visualized by spraying with anisaldehyde-H_2_SO_4_ reagent.

### Animal Material

The marine sponge *H. lachne* was collected from the Xisha Islands in the South China Sea in March 2013, and identified by Prof. Jin-He Li (Institute of Oceanology, Chinese Academy of Sciences, China). The voucher specimens of *H. lachne* (RM-2013) is deposited at Research Center for Marine Drugs, State Key Laboratory of Oncogenes and Related Genes, Department of Pharmacy, Renji Hospital, School of Medicine, Shanghai Jiao Tong University.

### Extraction and Isolation

The sponge *H. lachne* (1.2 kg, dry weight) was cut into small pieces and exhaustively extracted by percolation with 95% EtOH at room temperature to give 56.0 g extract, which was suspended in H_2_O and extracted sequentially with EtOAc to afford EtOAc-soluble extract (27.5 g). The EtOAc-soluble extract was dissolved in 90% aqueous MeOH, and extracted with petroleum ether to yield petroleum ether-soluble extract (15.2 g). The 90% aqueous MeOH phase was diluted to 60% MeOH with H_2_O, and extracted with CH_2_Cl_2_ to yield CH_2_Cl_2_-soluble extract (10.0 g). The CH_2_Cl_2_-soluble extract was subjected to column chromatography on silica gel with a gradient elution of MeOH in CH_2_Cl_2_ to give six fractions (A–F). Fraction C was subjected to column chromatography (CC) on Sephadex LH-20 eluting with CH_2_Cl_2_–MeOH (1:1) to afford three subfractions (C1–C3). Subfraction C2 was separated by CC on ODS to give nine subfractions (C2A–C2I) with 80% MeOH-H_2_O as elution. Fr. C2C was purified by reversed-phase semipreparative HPLC (65% MeCN/H_2_O, 2.0 mL/min, 250 nm) to obtain **3** (3.0 mg, t_R_ = 51.0 min). Fr. C2D was further purified by reversed-phase semipreparative HPLC (50% MeCN/H_2_O, 2.0 mL/min, 210 nm) to obtain **1** (2.4 mg, t_R_ = 15.2 min) and **2** (2.8 mg, t_R_ = 18.0 min).

### Chemical structure data

All compounds were ≥98% pure, which were supported by the NMR spectra of the compounds provided in the Supporting Information.

#### Hipposponlachnin A (**1**)

Colorless crystal; 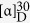 + 49 (*c* 0.11, MeOH); UV (MeOH) *λ*_max_ (log *ε*) 203 (3.37) nm; CD (MeCN) *λ*_max_ (Δ*ε*) 200 (+0.28), 308 (+0.16) nm; IR (KBr) *ν*_max_ 3280, 3167, 2943, 2922, 2861, 1732, 1453, 1410, 1377, 1339, 1306, 1252, 1147, 1122, 1076, 1045, 1025, 962, 942, 905, 858, 806, 672 cm^−1^; ^1^H and ^13^C NMR data see Table 1; HRESIMS *m/z* 343.2251 [M + Na]^+^ (calcd for C_20_H_32_O_3_Na, 343.2249).

#### Hipposponlachnin B (**2**)

Colorless crystal; 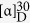 − 44 (*c* 0.24, MeOH); UV (MeOH) *λ*_max_ (log *ε*) 204 (3.47) nm; CD (MeCN) *λ*_max_ (Δ*ε*) 208 (−0.13), 308 (−0.25) nm; IR (KBr) *ν*_max_ 3375, 3331, 2953, 2926, 2900, 1726, 1704, 1456, 1412, 1377, 1360, 1329, 1263, 1240, 1171, 1130, 1088, 1032, 1009, 982, 957, 906, 874, 839, 804 cm^−1^; ^1^H and ^13^C NMR data see Table 1; HRESIMS *m/z* 343.2247 [M + Na]^+^ (calcd for C_20_H_32_O_3_Na, 343.2249).

#### (1R*,2E,4R*,7E,10S*,11S*,12R*)-10,18-diacetoxydolabella-2,7-dien-6-one (**3**)

Colorless oil; 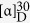 − 23 (*c* 0.15, MeOH); ^1^H NMR (CDCl_3_, 600 MHz) *δ* 6.05 (1H, s, H-7), 5.53 (1H, dd, *J* = 16.2 and 6.0 Hz, H-3), 4.99 (1H, d, *J* = 16.2 Hz, H-2), 4.81 (1H, dd, *J* = 10.2 and 3.0 Hz, H-10), 3.13 (1H, m, H-12), 2.69 (1H, dd, *J* = 11.4 and 3.6 Hz, H-5a), 2.55 (1H, m, H-4), 2.33 (3H, m, H-5b and H-9), 2.06 (3H, s, H-10-OAc), 2.01 (3H, br s, H-17), 1.95 (1H, m, H-13a), 1.92 (3H, s, H-18-OAc), 1.64 (1H, d, *J* = 10.2 Hz, H-11), 1.61 (3H, s, H-19), 1.49 (2H, m, H-13b and H-14a), 1.44 (1H, m, H-14b), 1.39 (3H, s, H-20), 1.02 (3H, d, *J* = 6.6 Hz, H-16), 0.86 (3H, s, H-15); ^13^C NMR (CDCl_3_, 150 MHz) *δ* 201.5 (C, C-6), 170.2 (C, C-10-OAc), 170.0 (C, C-18-OAc), 146.3 (C, C-8), 138.2 (CH, C-2), 132.1 (CH, C-3), 131.3 (CH, C-7), 84.7 (C, C-18), 71.9 (CH, C-10), 53.8 (CH, C-11), 51.1 (CH_2_, C-5), 47.8 (C, C-1), 44.9 (CH, C-12), 43.8 (CH_2_, C-9), 39.1 (CH_2_, C-14), 34.1 (CH, C-4), 26.7 (CH_3_, C-19), 26.2 (CH_2_, C-13), 23.3 (CH_3_, C-20), 23.0 (CH_3_, C-18-OAc), 21.5 (CH_3_, C-17), 21.0 (CH_3_, C-10-OAc), 18.3 (CH_3_, C-16), 17.5 (CH_3_, C-15); HRESIMS *m/z* 427.2451 [M + Na]^+^ (calcd for C_24_H_36_O_5_Na, 427.2460).

### X-ray Crystallographic Analysis of 1 and 2

Hipposponlachnins A (**1**) and B (**2**) were crystallized from MeOH at room temperature. The X-ray crystallographic data of them were measured on a Bruker Apex-II CCD diffractometer employing graphite monochromated Cu-K*α* radiation (*λ* = 1.54178 Å) at 140(2) K (operated in the *φ*-*ω* scan mode). The structures were solved by direct method using SHELXS-97 program and refined with full-matrix least-squares calculations on F^2^ using SHELXL-97. Crystallographic data for **1** and **2** have been deposited at the Cambridge Crystallographic Data Centre. Copies of these data can be obtained free of charge via the internet at www.ccdc.cam.ac.uk/conts/retrieving.html or on application to CCDC, 12 Union Road, Cambridge CB2 1EZ, UK [Tel: (+44) 1223-336-408; Fax: (+44) 1223-336-033; E-mail: deposit@ccdc.cam.ac.uk].

#### Crystallographic data of **1**

Colorless crystal, C_20_H_32_O_3_, *M*_r_ = 320.45, orthorhombic, space group *P*2_1_2_1_2_1_, *a* = 6.62210(10) Å, *b* = 15.2395(3) Å, *c* = 18.4069(3) Å, *α* = *β* = *γ* = 90°, *V* = 1857.58(6) Å^3^, *T* = 140 K, *Z* = 4, *D*_calcd_ = 1.146 g/cm^3^, crystal size 0.20 × 0.12 × 0.05 mm^3^, μ = 0.588 mm^−1^, F(000) = 704, 8888 reflections measured (7.53° < 2*θ* < 139.318°), 3177 unique (*R*_int_ = 0.0311, R_sigma_ = 0.0335). The final *R*_1_ value is 0.0354 [*I* > 2*σ*(*I*)] and *w*R_2_ = 0.0953 (all data). The goodness of fit on F^2^ was 1.060. Flack parameter = 0.13 (13). CCDC deposition number: 1501503.

#### Crystallographic data of **2**

Colorless crystal, C_20_H_32_O_3_, *M*_r_ = 320.45, monoclinic, space group *P*2_1_, *a* = 8.17720(10) Å, *b* = 9.71010(10) Å, *c* = 11.2700(2) Å, *α* = *γ* = 90°, *β* = 92.8110(10)°, *V* = 893.78(2) Å^3^, *T* = 140 K, *Z* = 2, *D*_calcd_ = 1.191 g/cm^3^, crystal size 0.20 × 0.10 × 0.02 mm^3^, μ = 0.611 mm^−1^, F(000) = 352, 6940 reflections measured (7.854° < 2*θ* < 139.132°), 2953 unique (*R*_int_ = 0.0248, R_sigma_ = 0.0310). The final *R*_1_ value is 0.0307 [*I* > 2*σ*(*I*)] and *w*R_2_ = 0.0818 (all data). The goodness of fit on F^2^ was 1.066. Flack parameter = −0.03 (9). CCDC deposition number: 1501504.

### Anti-allergic assay

Cell viability were determined using the MTT method. RBL-2H3 cells were plated into a 96-well plate at 5 × 10^5^ cells per well (100 *μ*L/well) for 24 h. Subsequently, cells were incubated with hipposponlachnins A (**1**) and B (**2**) for 24 hours and medium was replaced with MTT solution (250 *μ*g/mL) and incubated at 37 °C for 4 h. The medium was carefully discarded and formazan was resuspended in 150 *μ*L of dimethyl sulfoxide (DMSO). The absorbance was measured at 490 nm using a microplate reader. Values measured from untreated cells were considered to represent 100% viability. RBL-2H3 cells were seeded in a 24-well plate at 5 × 10^5^ cells per well, and sensitized with dinitrophenyl (DNP)-specific IgE (DNP-IgE) (1 *μ*g/mL) at 37 °C overnight. DNP-IgE-sensitized cells were preincubated with hipposponlachnins A (**1**) and B (**2**) for 30 min, and then stimulated with DNP-BSA for 1.5 h. To measure the activity of *β*-hexosaminidase released from the cells, cultured media were centrifuged (17,000 g, 10 min) at 4 °C. The supernatant (50 *μ*L) was mixed with 50 *μ*L of 0.1 M sodium citrate buffer (pH 4.5) containing 10 mM 4-nitrophenyl N-acetyl-*β*-D-glucosaminide in a 96-well plate, and then incubated for 90 min at 37 °C. The absorbance was measured at 405 nm after terminating the reaction by 0.2 M glycine (pH 10.0). To measure Interleukin-4 (IL-4) and Leukotriene B_4_ (LTB_4_) level in cultured media, all cultured media were centrifuged at 4 °C, and hipposponlachnins A (**1**) and B (**2**) were stored at −80 °C until assay. IL-4 and LTB4 were quantified using an ELISA kit according to the manufacturer’s instructions. The data were analyzed using a one-way ANOVA followed by Dunnett’s Multiple Comparison Test with GraphPad Prism software (GraphPad Prism version 5.01 for Windows, San Diego, CA, USA). The values are expressed as the means ± SD. The differences with p < 0.05 were considered significant.

## Additional Information

**How to cite this article**: Hong, L.-L. *et al*. Unusual Anti-allergic Diterpenoids from the Marine Sponge *Hippospongia lachne.* *Sci. Rep.* **7**, 43138; doi: 10.1038/srep43138 (2017).

**Publisher's note:** Springer Nature remains neutral with regard to jurisdictional claims in published maps and institutional affiliations.

## Supplementary Material

Supplementary Information

## Figures and Tables

**Table 1:** ^1^H and ^13^C NMR Data for **1** and **2** (500, 125 MHz, CDCl_3_, TMS, *δ* ppm).

position	1		2	
	*δ*_C_, type	*δ*_H_, mult (*J* in Hz)	*δ*_C_, type	*δ*_H_, mult (*J* in Hz)
1	45.9, C		43.1, C	
2	54.01, CH	1.84, d (7.5)	50.4, CH	2.10, d (7.0)
3	41.0, CH	2.47, t (7.5)	39.2, CH	2.90, q (7.0)
4	34.2, CH	2.10, m	32.3, CH	2.33, m
5*α*	47.1, CH_2_	2.00, d (19.0)	46.0, CH_2_	2.17, m
5*β*		2.75, dd (19.0, 7.5)		2.34, m
6	220.6, C		220.4, C	
7	53.98, CH	2.34, d (7.5)	56.1, CH	2.32, m
8	39.3, C		38.0, C	
9*α*	48.8, CH_2_	1.52, dd (21.0, 10.0)	40.9, CH_2_	1.65, d (15.0)
9*β*		2.10, m		2.15, m
10	67.2, CH	3.72, td (10.0, 4.0)	70.2, CH	3.92, dd (9.5, 7.5)
11	50.3, CH	1.53, dd (10.0, 4.0)	49.4, CH	1.54, t (9.5)
12	51.2, CH	2.10, m	52.8, CH	2.03, m
13*α*	26.0, CH_2_	1.28, m	27.2, CH_2_	1.31, m
13*β*		1.94, m		1.96, m
14*α*	33.1, CH_2_	1.28, m	42.3, CH_2_	1.22, d (11.0)
14*β*		1.24, m		1.60, dd (11.0, 8.5)
15	22.7, CH_3_	0.85, s	19.6, CH_3_	0.93, s
16	21.1, CH_3_	0.97, d (7.0)	15.5, CH_3_	1.01, d (6.0)
17	26.5, CH_3_	1.11, s	26.2, CH_3_	1.12, s
18	72.7, C		73.2, C	
19	30.8, CH_3_	1.28, s	30.9, CH_3_	1.27, s
20	23.0, CH_3_	1.24, s	23.0, CH_3_	1.19, s

## References

[b1] Galli S. J., Tsai M. & Piliponsky A. M. The development of allergic inflammation. Nature 454, 445–454 (2008). 18650915 10.1038/nature07204 PMC3573758

[b2] Tewtrakul S. & Subhadhirasakul S. Anti-allergic activity of some selected plants in the Zingiberaceae family. J. Ethnopharmacol. 109, 535–538 (2007). 16978816 10.1016/j.jep.2006.08.010

[b3] Ho C., Choi E. J., Yoo G. S., Kim K. M. & Ryu S. Y. Desacetylmatricarin, an Anti-Allergic Component from *Taraxacum platycarpum*. Planta Med. 64, 577–578 (1998). 9741305 10.1055/s-2006-957520

[b4] Hwang D. . Effects of Flavone Derivatives on Antigen-Stimulated Degranulation in RBL-2H3 Cells. Chem. Biol. Drug Des. 81, 228–237 (2013). 23035634 10.1111/cbdd.12067

[b5] Newman D. J. & Cragg G. M. Natural Products as Sources of New Drugs from 1981 to 2014. J. Nat. Prod. 79, 629–661 (2016). 26852623 10.1021/acs.jnatprod.5b01055

[b6] Mehbub M., Lei J., Franco C. & Zhang W. Marine Sponge Derived Natural Products between 2001 and 2010: Trends and Opportunities for Discovery of Bioactives. Mar. Drugs 12, 4539–4577 (2014). 25196730 10.3390/md12084539 PMC4145330

[b7] Piao S. J. . Hippolachnin A, a New Antifungal Polyketide from the South China Sea Sponge *Hippospongia lachne*. Org. Lett. 15, 3526–3529 (2013). 23829334 10.1021/ol400933x

[b8] Oda T., Wang W., Ukai K., Nakazawa T. & Mochizuki M. A Sesquiterpene Quinone, 5-Epi-smenospongine, Promotes TNF-α Production in LPS-stimulated RAW 264.7 Cells. Mar. Drugs 5, 151–156 (2007). 18463729 10.3390/md504151 PMC2365696

[b9] Piao S. J. . Hippolides A-H, Acyclic Manoalide Derivatives from the Marine Sponge *Hippospongia lachne*. J. Nat. Prod. 74, 1248–1254 (2011). 21548579 10.1021/np200227s

[b10] Rifai S., Fassouane A., Kijjoa A. & Van Soest R. Antimicrobial activity of untenospongin B, a metabolite from the marine sponge *Hippospongia communis* collected from the Atlantic Coast of Morocco. Mar. Drugs 2, 147–153 (2004).

[b11] Craig K. S. . Novel sesterterpenoid and norsesterterpenoid RCE-protease inhibitors isolated from the marine sponge *Hippospongia* sp. Tetrahedron Lett. 43, 4801–4804 (2002).

[b12] Ishiyama H., Ishibashi M., Ogawa A. & Yoshida S. & Kobayashi, J. i. Taurospongin A, novel acetylenic fatty acid derivative inhibiting DNA polymerase β and HIV reverse transcriptase from sponge *Hippospongia* sp. J. Org. Chem. 62, 3831–3836 (1997).

[b13] Madaio A., Notaro G., Piccialli V. & Sica D. Minor 5, 6-secosterols from the marine sponge Hippospongia communis. Isolation and synthesis of (7Z, 22E, 24R)-24-methyl-5, 6-secocholesta-7, 22-diene-3β, 5β, 6-triol. J. Nat. Prod. 53, 565–572 (1990).

[b14] Umeyama A., Shoji N., Arihara S., Ohizumi Y. & Kobayashi J. Untenospongins A and B, novel furanoterpenes with coronary vasodilating activity from the okinawan marine sponge *Hippospongia* sp. Aust. J. Chem. 42, 459–462 (1989).

[b15] Lu Q. & Faulkner D. J. Three Dolabellanes and a Macrolide from the Sponge *Dysidea* sp. from Palau. J. Nat. Prod. 61, 1096–1100 (1998). 9748373 10.1021/np980134e

[b16] Costantino V. . A New Cytotoxic Diterpene with the Dolabellane Skeleton from the Marine Sponge *Sigmosceptrella quadrilobata*. Eur. J. Org. Chem. 1999, 227–230 (1999).

[b17] Cao R. . Diterpene cyclases and the nature of the isoprene fold. Proteins: Structure, Function, and Bioinformatics 78, 2417–2432 (2010). 10.1002/prot.22751 PMC3805035 20602361

[b18] Huang X. . Asperterpenoid A, a New Sesterterpenoid as an Inhibitor of Mycobacterium tuberculosis Protein Tyrosine Phosphatase B from the Culture of *Aspergillus* sp. 16–5c. Org. Lett. 15, 721–723 (2013). 23360543 10.1021/ol303549c

[b19] Lou H. . Vulgarisin A, a New Diterpenoid with a Rare 5/6/4/5 Ring Skeleton from the Chinese Medicinal Plant *Prunella vulgaris*. Org. Lett. 16, 2696–2699 (2014). 24796480 10.1021/ol5009763

[b20] Liang L. F. . Unprecedented Diterpenoids as a PTP1B Inhibitor from the Hainan Soft Coral *Sarcophyton trocheliophorum Marenzeller*. Org. Lett. 15, 274–277 (2013). 23273218 10.1021/ol303110d

[b21] Fink A. L. & Hay G. W. The enzymic deacylation of esterified mono- and di-saccharides. IV. The products of esterase-catalyzed deacetylations. Can. J. Biochem. 47, 353–359 (1969). 5778218 10.1139/o69-053

[b22] Park D. K., Choi W. S. & Park H.-J. Antiallergic Activity of Novel Isoflavone Methyl-glycosides from *Cordyceps militaris* Grown on Germinated Soybeans in Antigen-Stimulated Mast Cells. J. Agric. Food Chem. 60, 2309–2315 (2012). 22296272 10.1021/jf205199j

