# Supplementary material for: Unusual Anti-allergic Diterpenoids from the Marine Sponge Hippospongia lachne
Source: Sci Rep. 2017 Feb 22;7:43138. doi: 10.1038/srep43138 (PMC5320514; doi:10.1038/srep43138)
Supplement: Supplementary Information [file srep43138-s1.pdf]

Supplementary Information

Unusual Anti-allergic Diterpenoids from the Marine Sponge *Hippospongia lachne*

Li-Li Hong[1,+], Hao-Bing Yu[1,+], Jie Wang[2], Wei-Hua Jiao[1], Bao-Hui Cheng[3], Fan Yang[1], Yong-Jun Zhou[1], Bin-Bin Gu[1], Shao-Jiang Song[2,*] & Hou-Wen Lin[1,*]

[1]Research Center for Marine Drugs, State Key Laboratory of Oncogenes and Related Genes, Department of Pharmacy, Renji Hospital, School of Medicine, Shanghai Jiao Tong University, Shanghai 200127, P. R. China

[2]Department of Natural Products Chemistry, Shenyang Pharmaceutical University, Shenyang 110016, P. R. China

[3]Shenzhen Key Laboratory of ENT, Longgang ENT hospital & Institute of ENT, Shenzhen 518172, P. R. China

[*]Corresponding author: Hou-Wen Lin, franklin67@126.com; Shao-Jiang Song, songsj99@163.com

[+]These authors contributed equally to this work

Table of Contents

No.		Content	Page
1	**Figure S1.**	^{1}H NMR (500 MHz, $CDCl_3$) spectrum of hipposponlachnin A (**1**)	S3
2	**Figure S2.**	^{13}C NMR (125 MHz, $CDCl_3$) spectrum of hipposponlachnin A (**1**)	S4
3	**Figure S3.**	DEPT135 spectrum of hipposponlachnin A (**1**) in $CDCl_3$	S5
4	**Figure S4.**	HSQC spectrum of hipposponlachnin A (**1**) in $CDCl_3$	S6
5	**Figure S5.**	COSY spectrum of hipposponlachnin A (**1**) in $CDCl_3$	S7
6	**Figure S6.**	HMBC spectrum of hipposponlachnin A (**1**) in $CDCl_3$	S8
7	**Figure S7.**	NOESY spectrum of hipposponlachnin A (**1**) in $CDCl_3$	S9
8	**Figure S8.**	HRESIMS of hipposponlachnin A (**1**)	S10
9	**Figure S9.**	UV spectrum of hipposponlachnin A (**1**) in MeOH	S11
10	**Figure S10.**	IR spectrum of hipposponlachnin A (**1**)	S12
11	**Figure S11.**	^{1}H NMR (500 MHz, $CDCl_3$) spectrum of hipposponlachnin B (**2**)	S13
12	**Figure S12.**	^{13}C NMR (125 MHz, $CDCl_3$) spectrum of hipposponlachnin B (**2**)	S14
13	**Figure S13.**	DEPT135 spectrum of hipposponlachnin B (**2**) in $CDCl_3$	S15
14	**Figure S14.**	HSQC spectrum of hipposponlachnin B (**2**) in $CDCl_3$	S16
15	**Figure S15.**	COSY spectrum of hipposponlachnin B (**2**) in $CDCl_3$	S17
16	**Figure S16.**	HMBC spectrum of hipposponlachnin B (**2**) in $CDCl_3$	S18
17	**Figure S17.**	NOESY spectrum of hipposponlachnin B (**2**) in $CDCl_3$	S19
18	**Figure S18.**	HRESIMS of hipposponlachnin B (**2**)	S20
19	**Figure S19.**	UV spectrum of hipposponlachnin B (**2**) in MeOH	S21
20	**Figure S20.**	IR spectrum of hipposponlachnin B (**2**)	S22
21	**Figure S21.**	CD spectra of hipposponlachnin A **(1)** and B (**2**) in MeCN.	S23
22	**Figure S22.**	ESIMS of **3**.	S24
23	**Figure S23.**	^{1}H NMR (600 MHz, $CDCl_3$) spectrum of **3**	S25
24	**Figure S24.**	^{13}C NMR (150 MHz, $CDCl_3$) spectrum of **3**	S26
25	**Figure S25.**	DEPT135 spectrum of **3** in $CDCl_3$	S27
26	**Figure S26.**	Proposed mechanism of the [2 + 2] cycloaddition of **3** and its 7,8-Z isomer	S28
27	**Table S1.**	Crystal data and structure refinement for hipposponlachnin A (**1**)	S29
28	**Table S2.**	Crystal data and structure refinement for hipposponlachnin B (**2**)	S30
29		CheckCIFPLATON report of hipposponlachnin A (**1**)	S31
30		CheckCIFPLATON report of hipposponlachnin B (**2**)	S33

Figure S1. ^{1}H NMR (500 MHz, $CDCl_3$) spectrum of hipposponlachnin A (**1**)

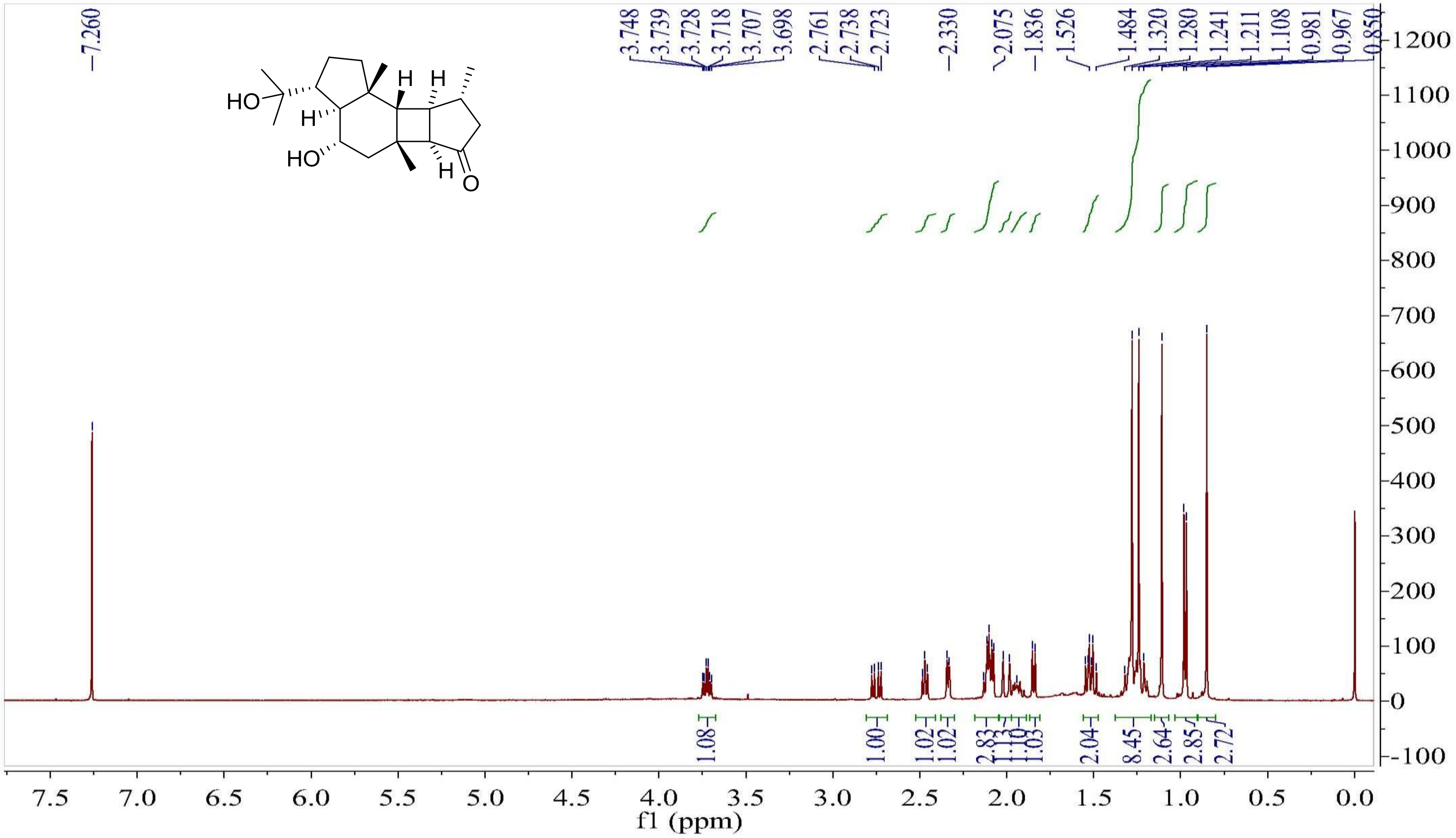

Figure S2. ^{13}C NMR (125 MHz, $CDCl_3$) spectrum of hipposponlachnin A (**1**)

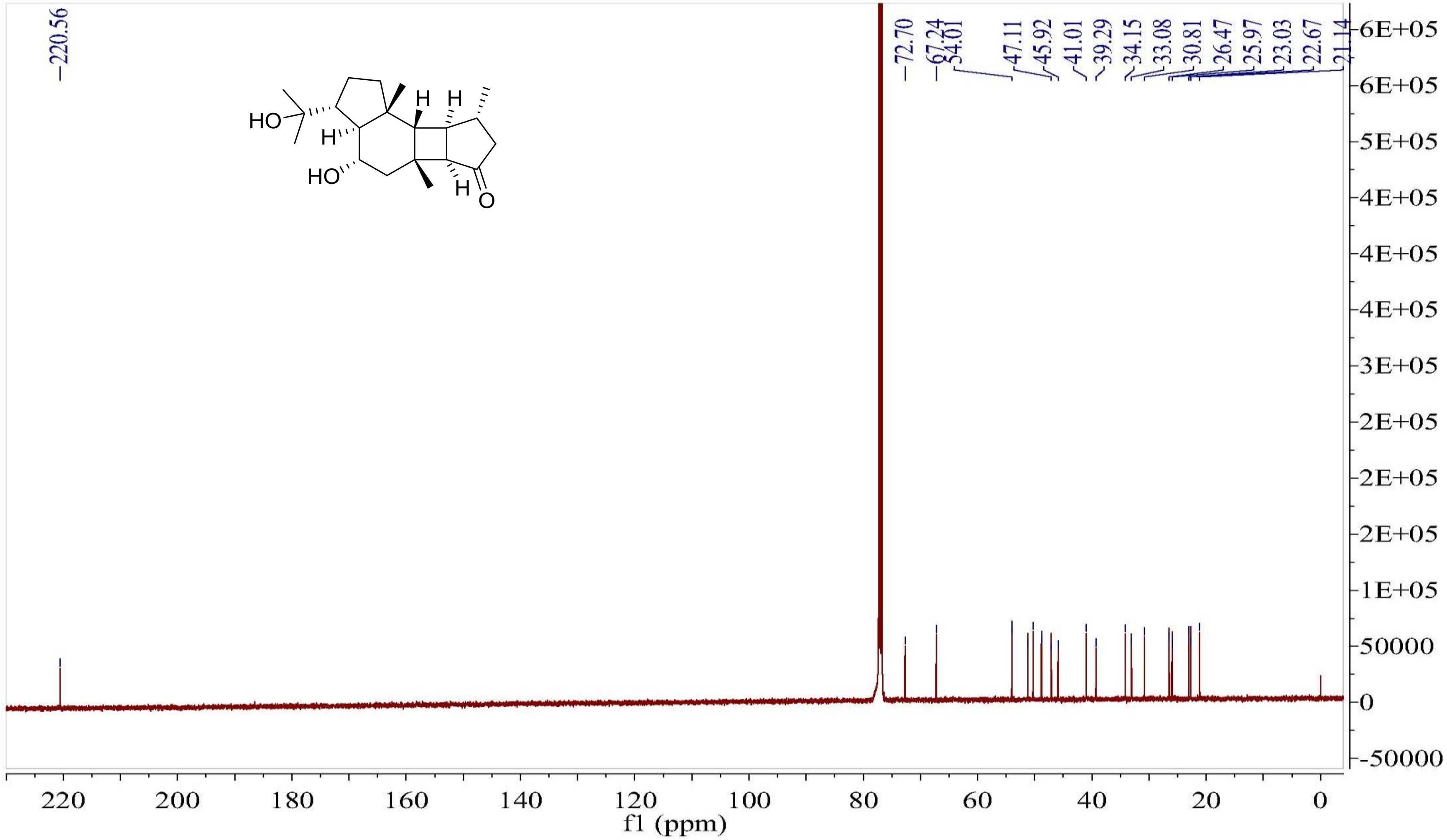

Figure S3. DEPT135 spectrum of hipposponlachnin A (**1**) in $CDCl_3$

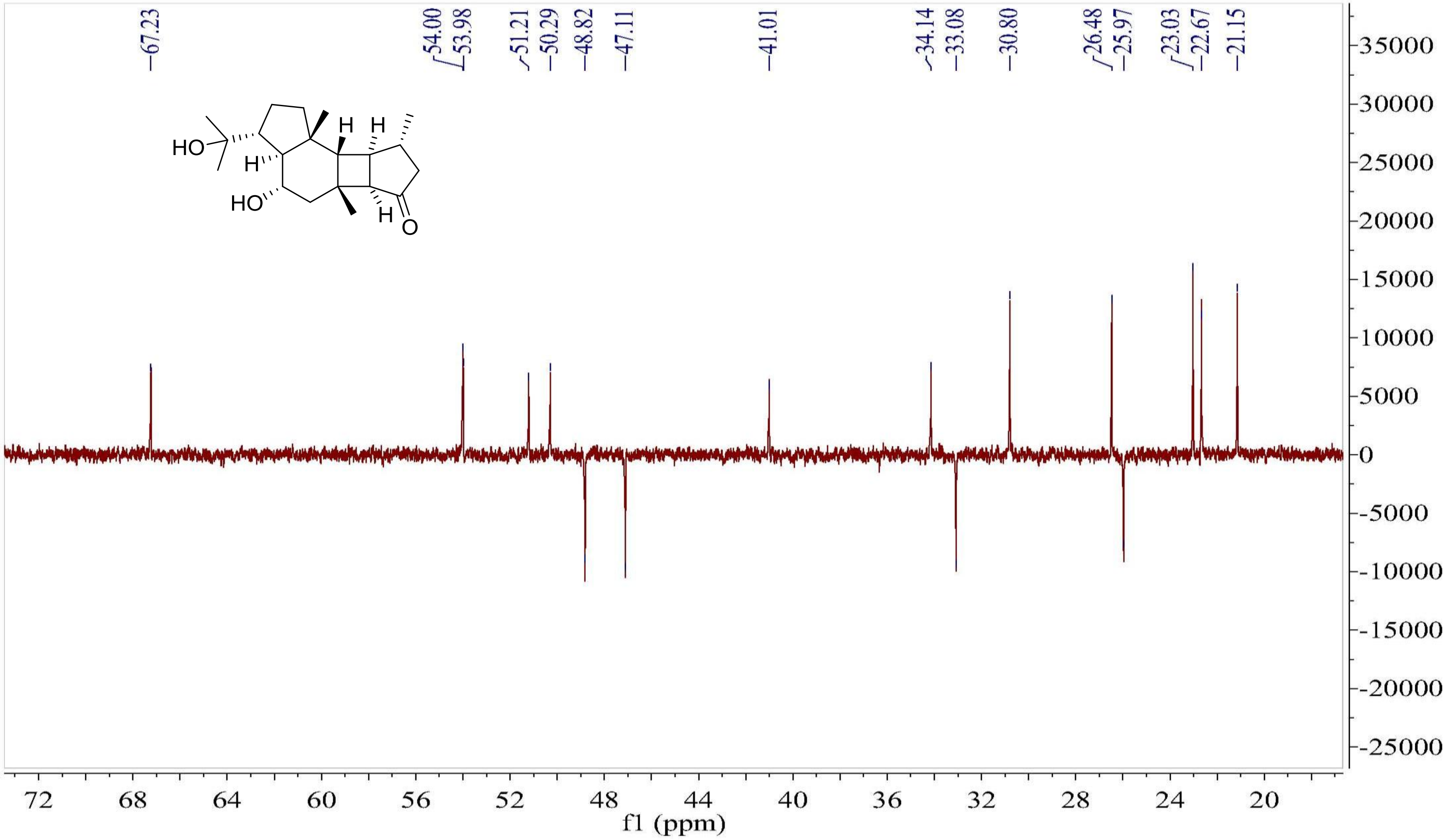

Figure S4. HSQC spectrum of hipposponlachnin A (**1**) in $CDCl_3$

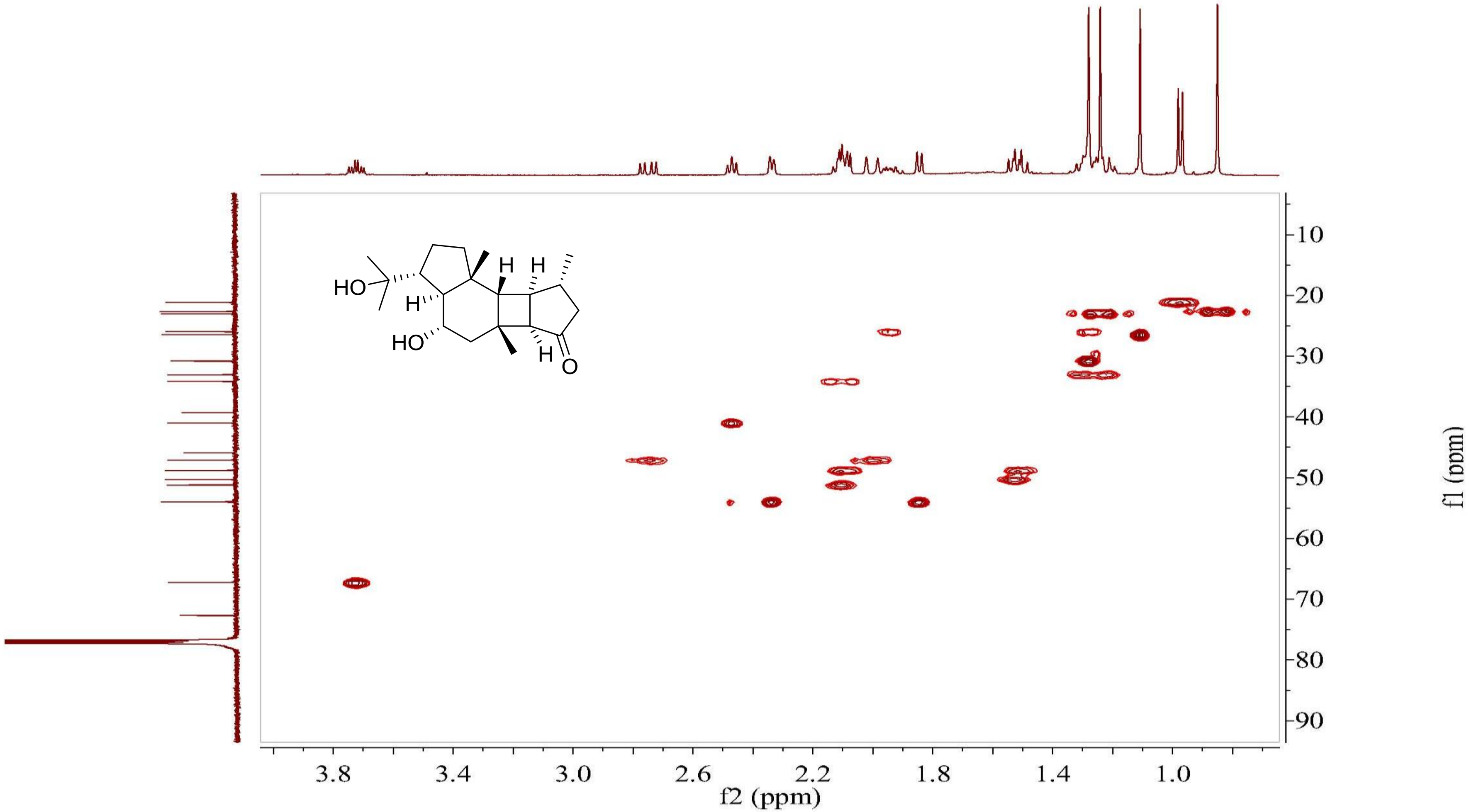

Figure S5. COSY spectrum of hipposponlachnin A (**1**) in $CDCl_3$

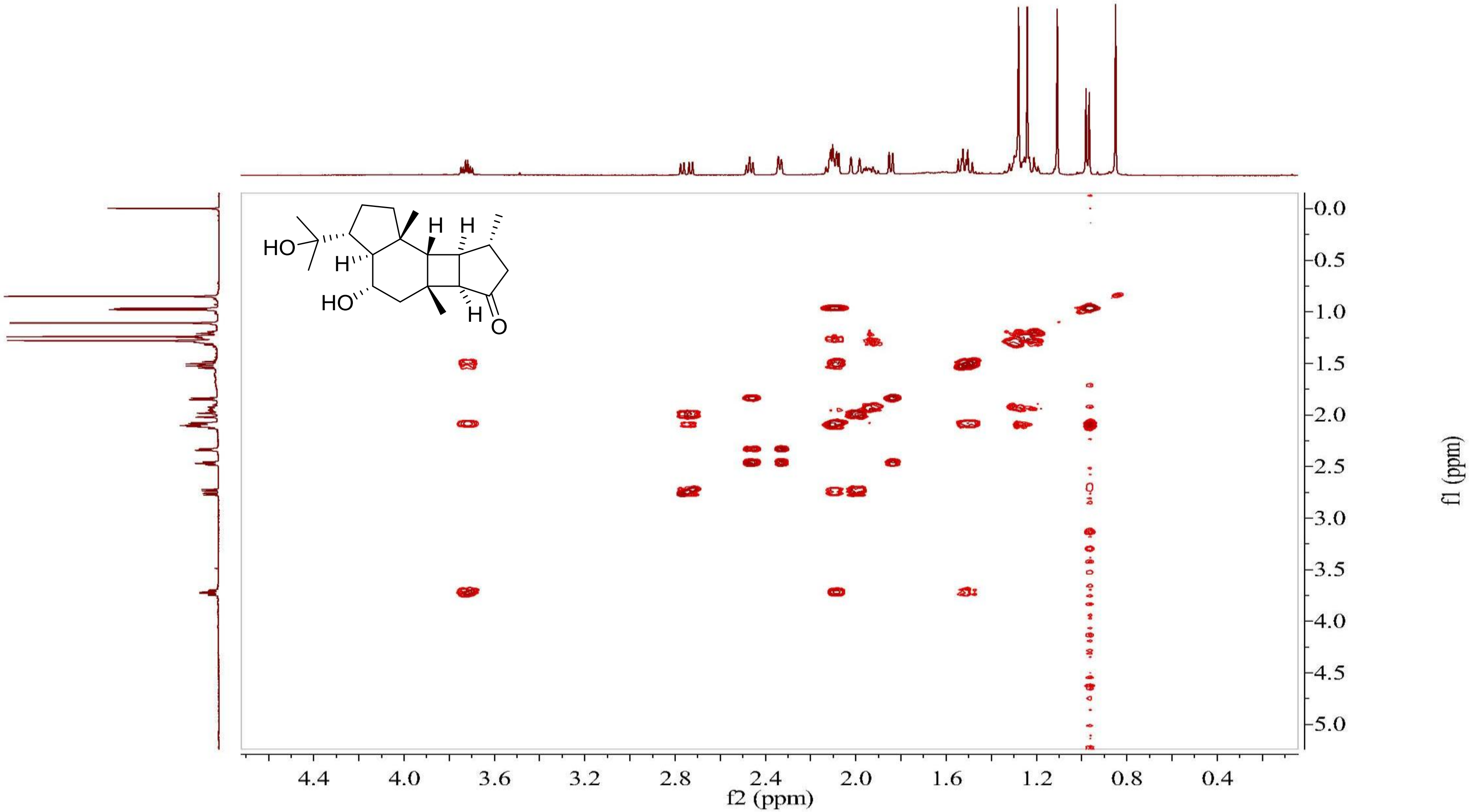

Figure S6. HMBC spectrum of hipposponlachnin A (**1**) in $CDCl_3$

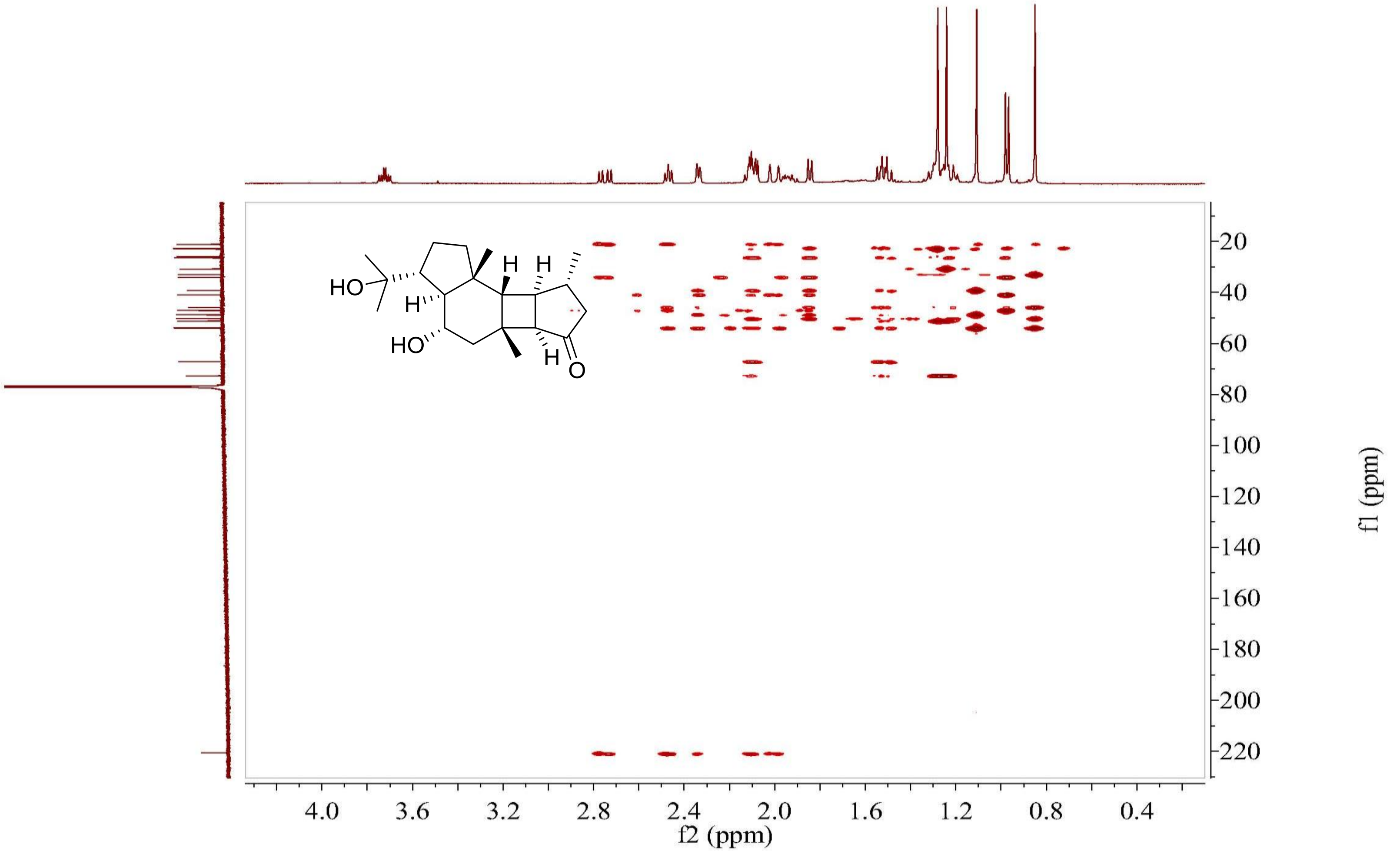

Figure S7. NOESY spectrum of hipposponlachnin A (**1**) in $CDCl_3$

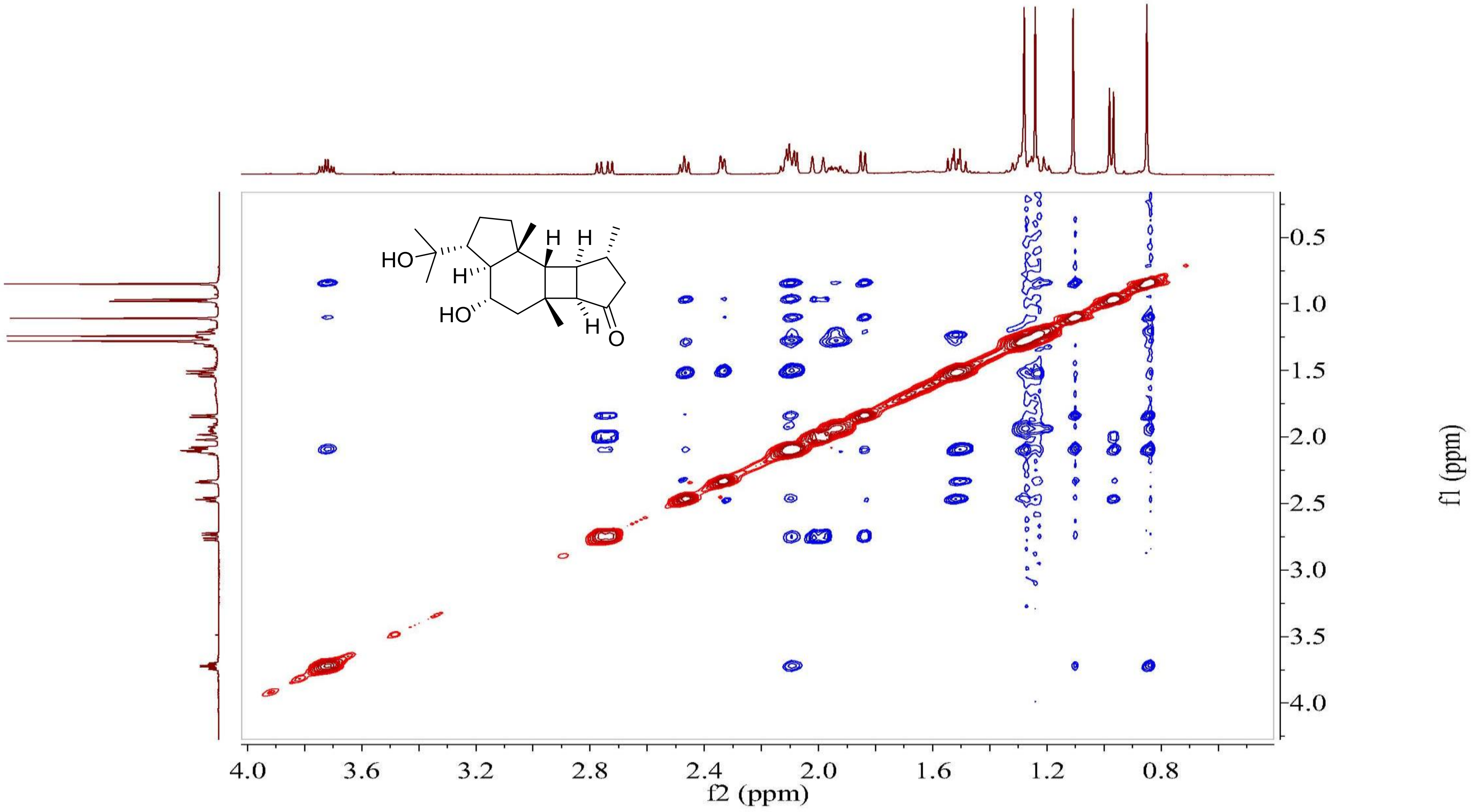

Figure S8. HRESIMS of hipposponlachnin A (**1**)

Tolerance = 10.0 PPM / DBE: min = -1.5, max = 50.0
Selected filters: None

Monoisotopic Mass, Even Electron Ions
8 formula(e) evaluated with 1 results within limits (up to 50 closest results for each mass)
Elements Used:
C: 10-22 H: 10-40 O: 1-4 Na: 1-1

SIPI Q-Tof micro 15-Jan-2014,15:48:14
hll-d246 M.W=320 YA019
WQ14-024H 21 (0.725) AM (Cen,4, 80.00, Ar,5000.0,335.08,0.70); Sm (Mn, 2x3.00); Cm (10:21) TOF MS ES+

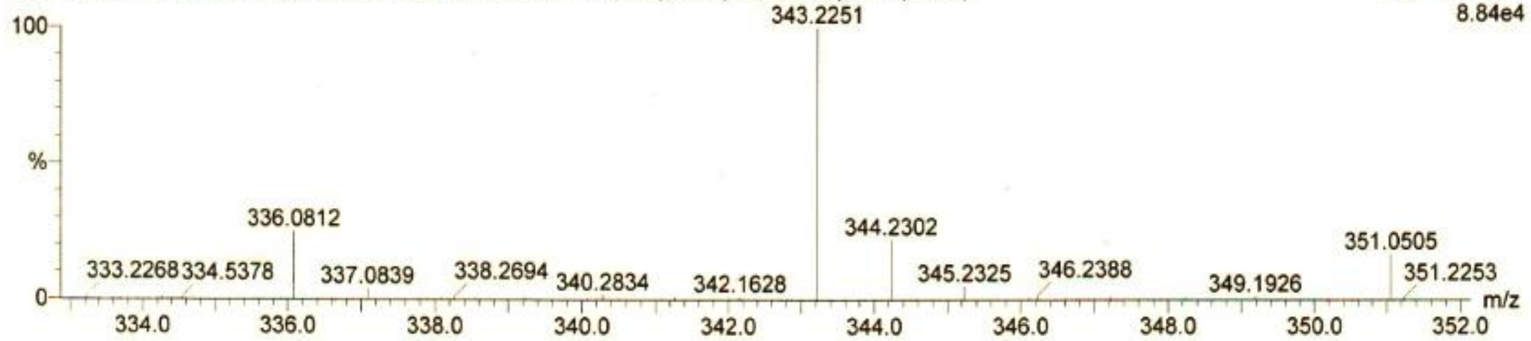

Minimum:	62.00				-1.5		
Maximum:	100.00		5.0	10.0	50.0		
Mass	RA	Calc. Mass	mDa	PPM	DBE	i-FIT	Formula
343.2251	100.00	343.2249	0.2	0.6	4.5	295.3	C20 H32 O3 Na

Figure S9. UV spectrum of hipposponlachnin A (**1**) in MeOH

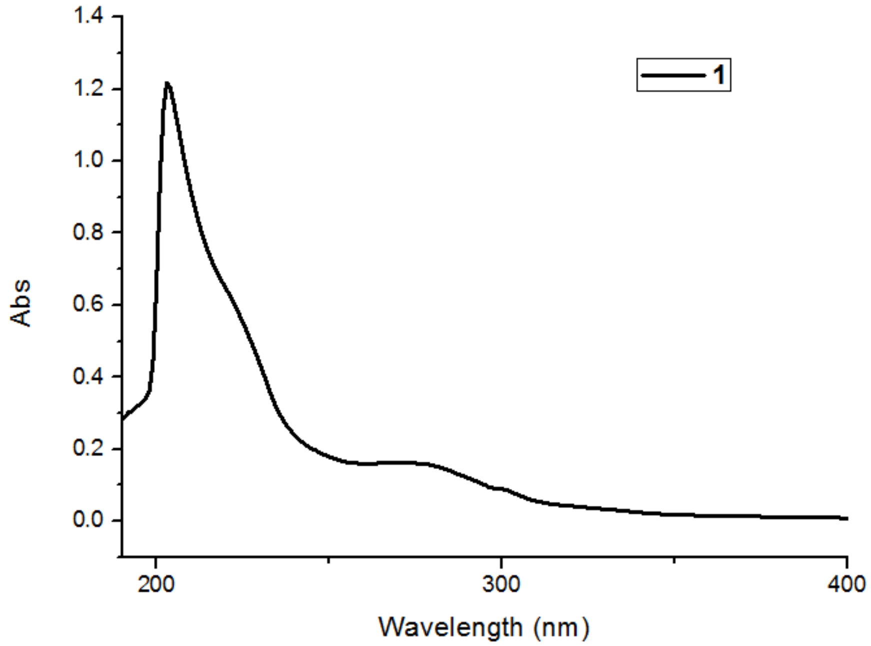

Figure S10. IR spectrum of hipposponlachnin A (**1**)

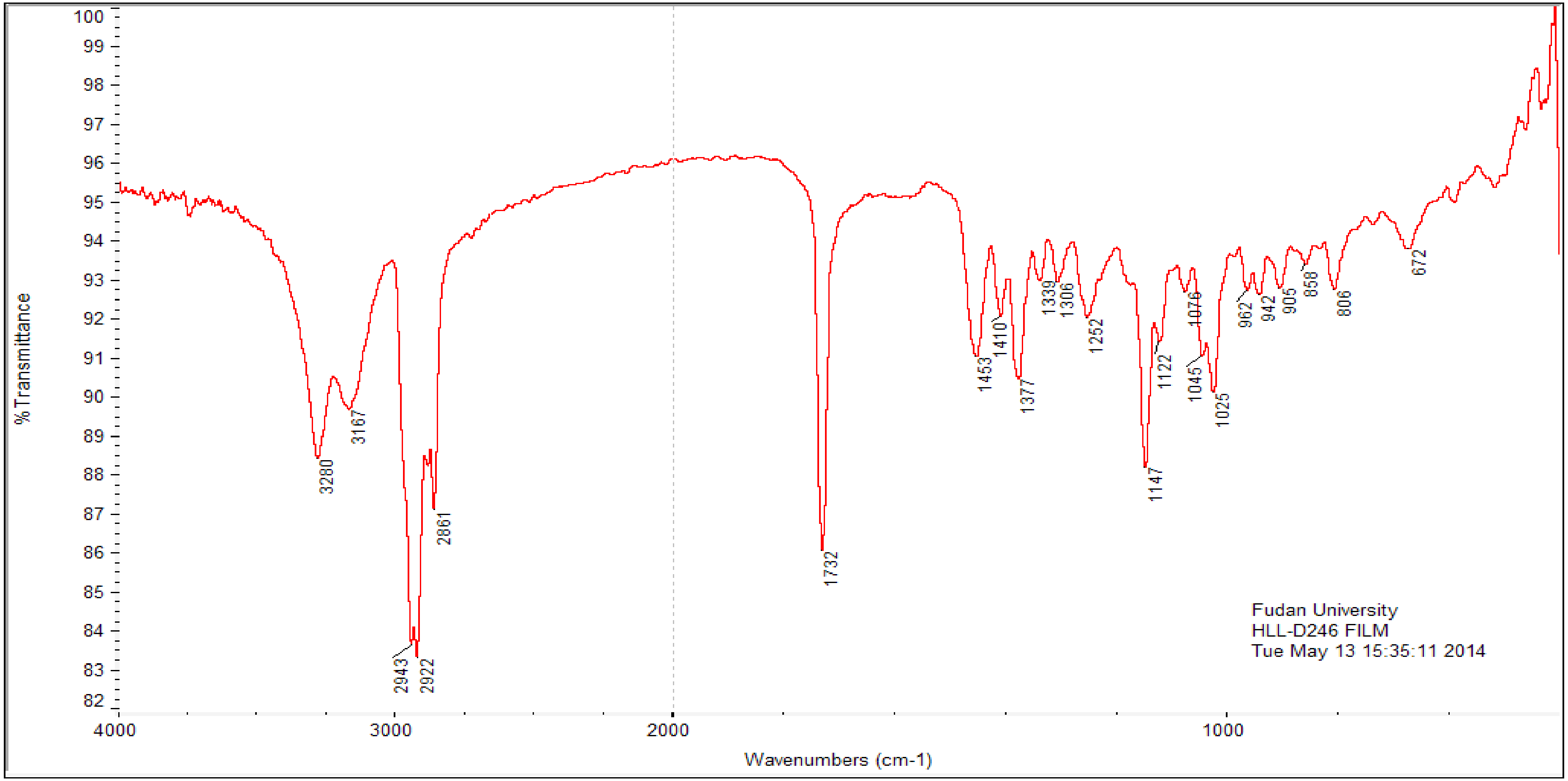

Figure S11. ^{1}H NMR (500 MHz, $CDCl_3$) spectrum of hipposponlachnin B (**2**)

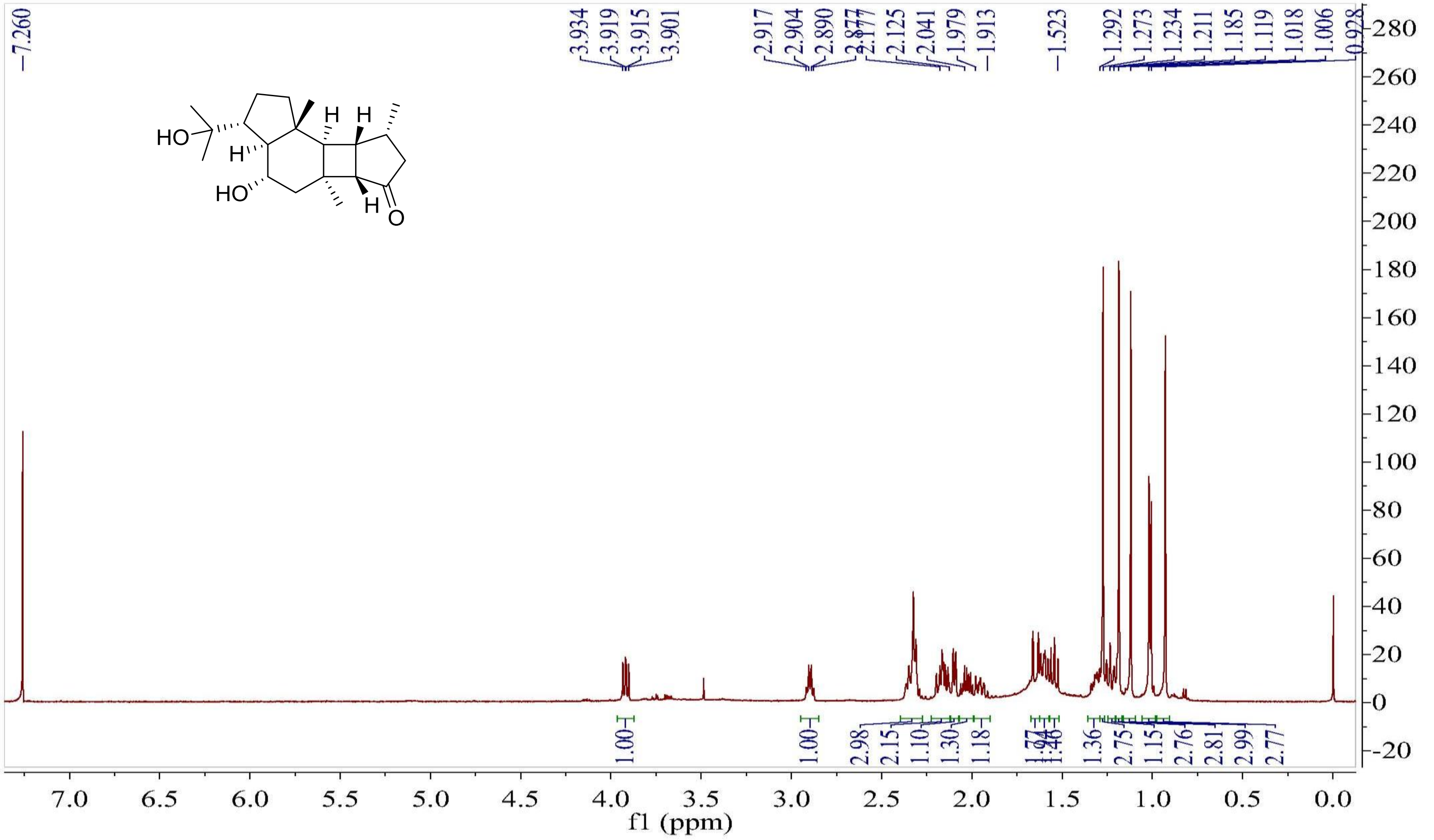

Figure S12. ^{13}C NMR (125 MHz, $CDCl_3$) spectrum of hipposponlachnin B (**2**)

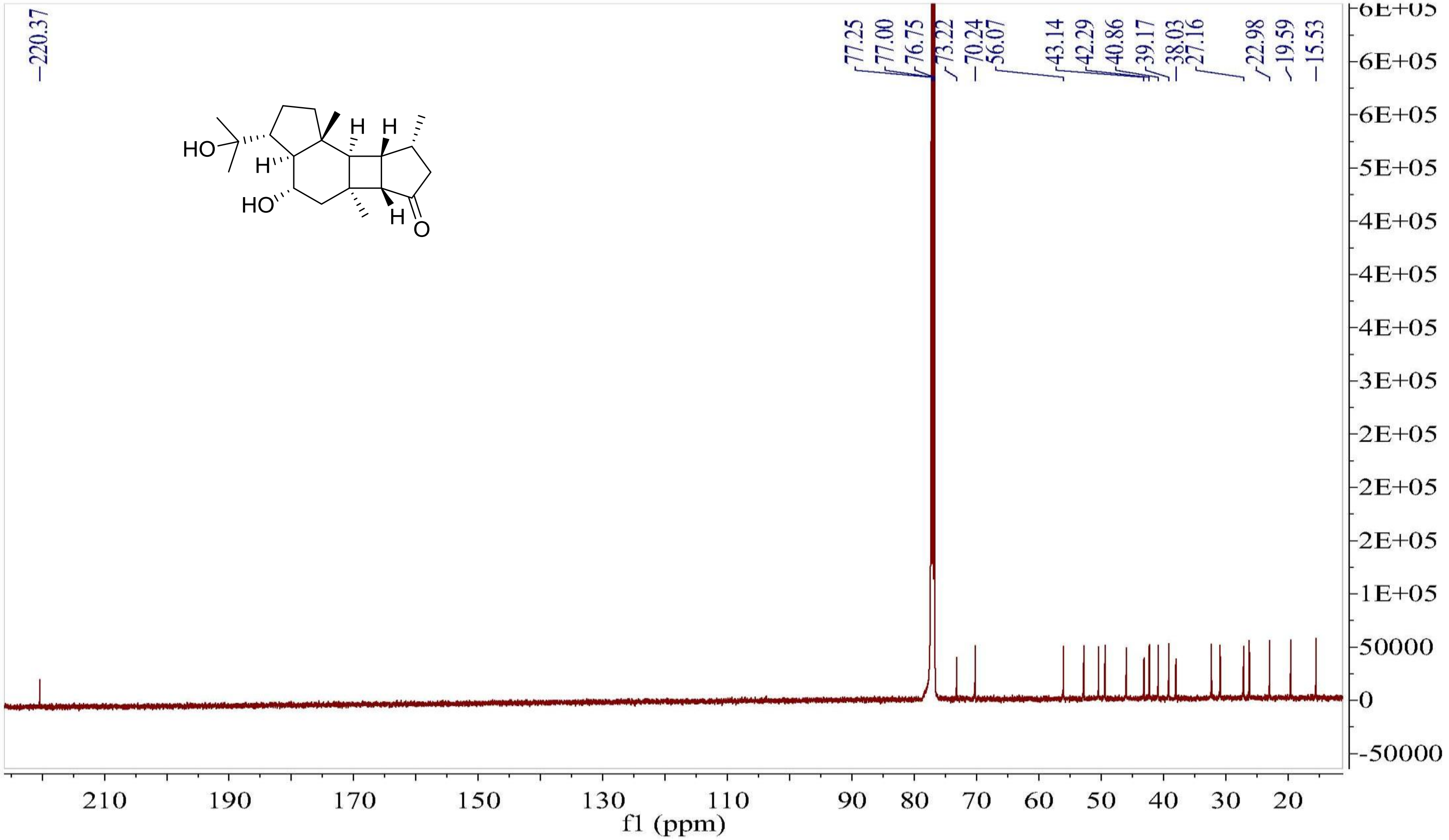

Figure S13. DEPT135 spectrum of hipposponlachnin B (**2**) in $CDCl_3$

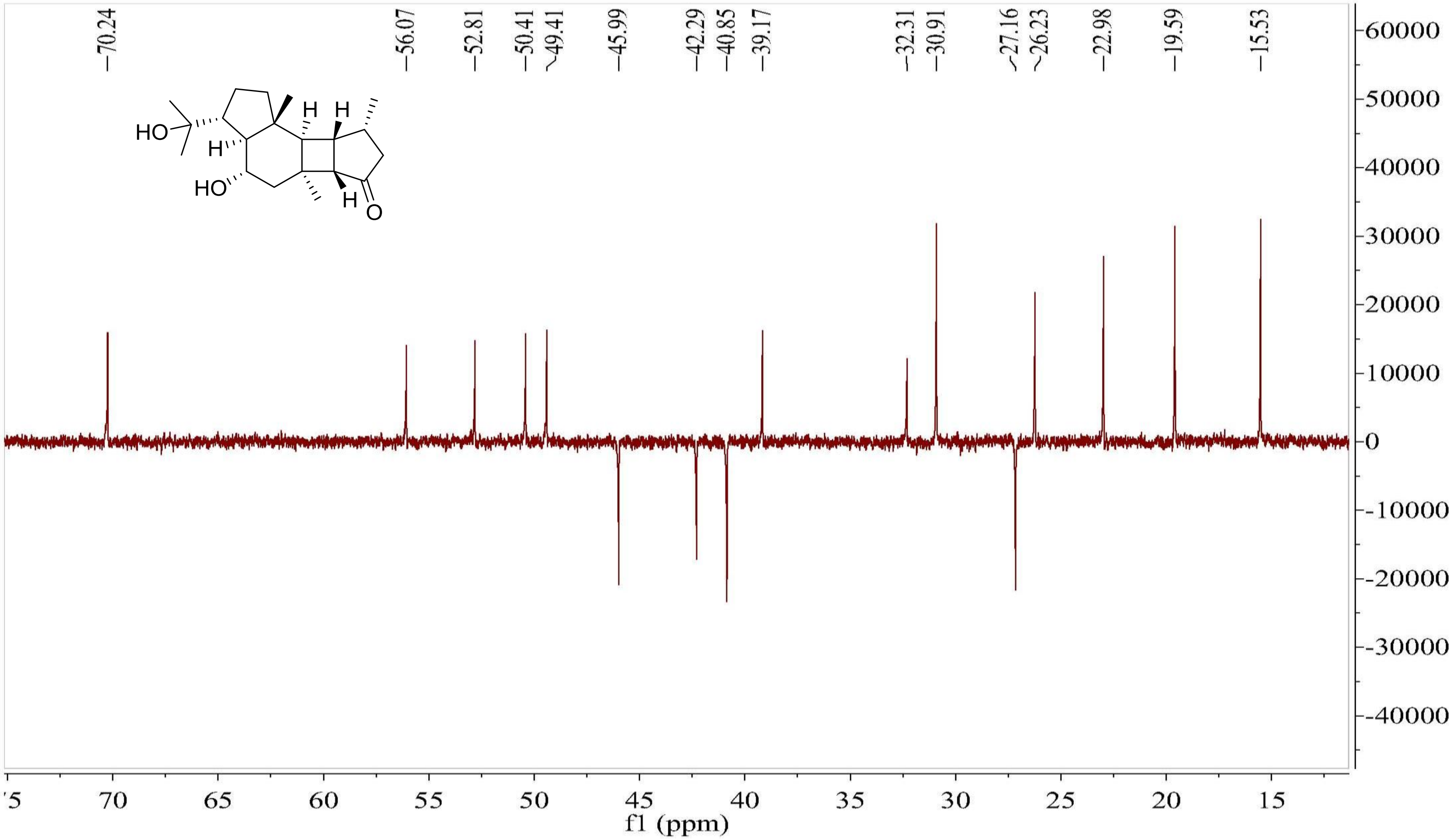

Figure S14. HSQC spectrum of hipposponlachnin B (**2**) in $CDCl_3$

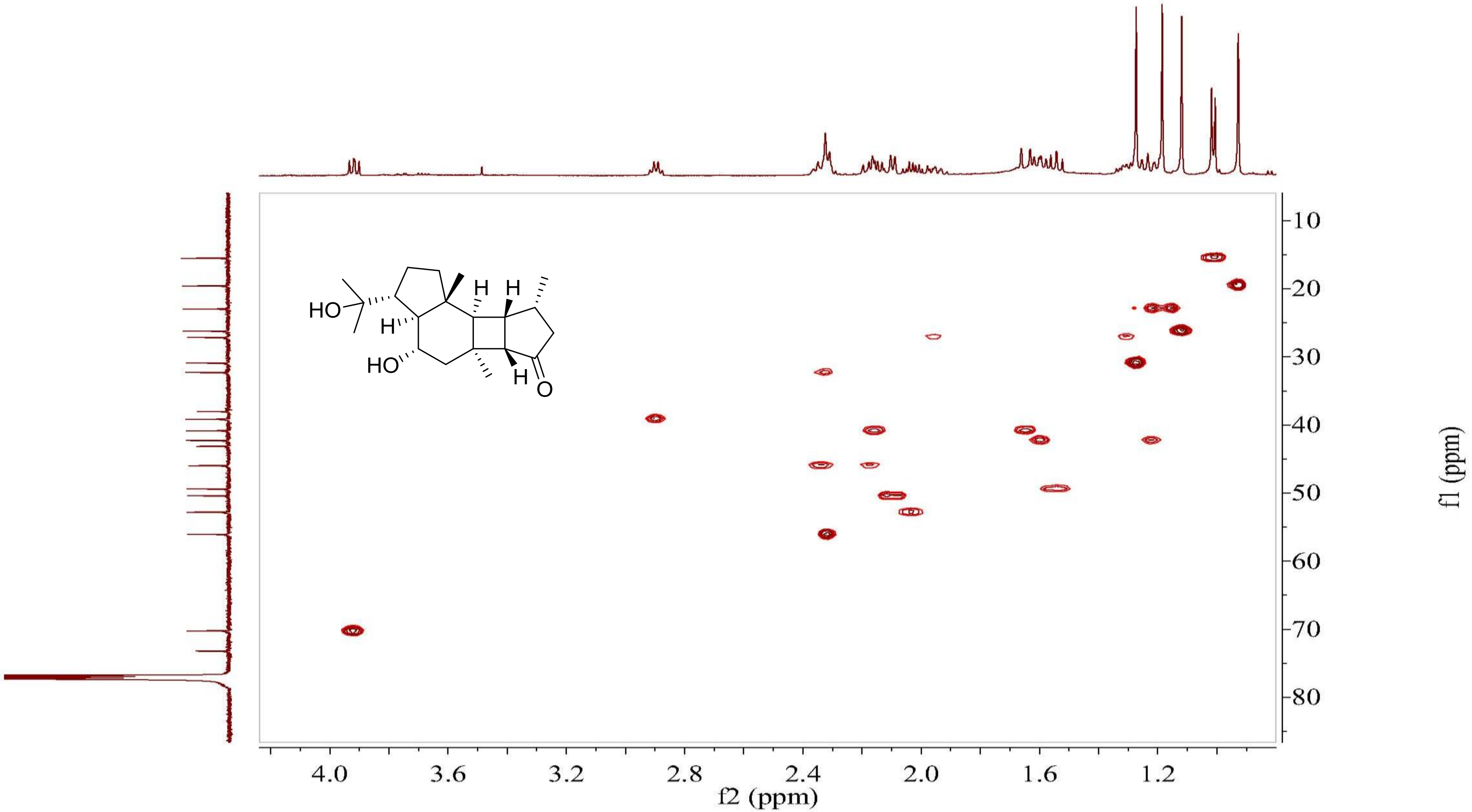

Figure S15. COSY spectrum of hipposponlachnin B (**2**) in $CDCl_3$

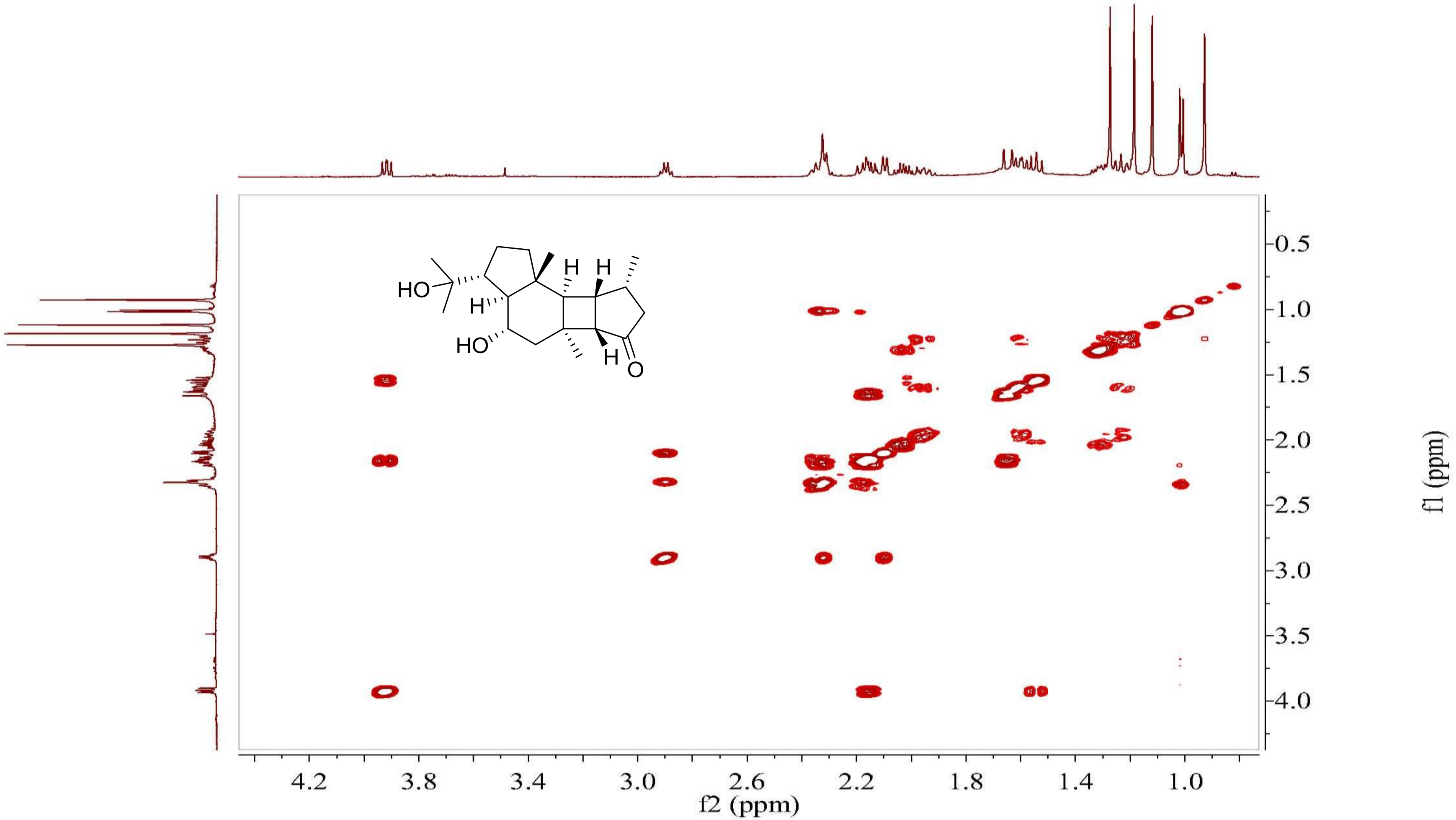

Figure S16. HMBC spectrum of hipposponlachnin B (**2**) in $CDCl_3$

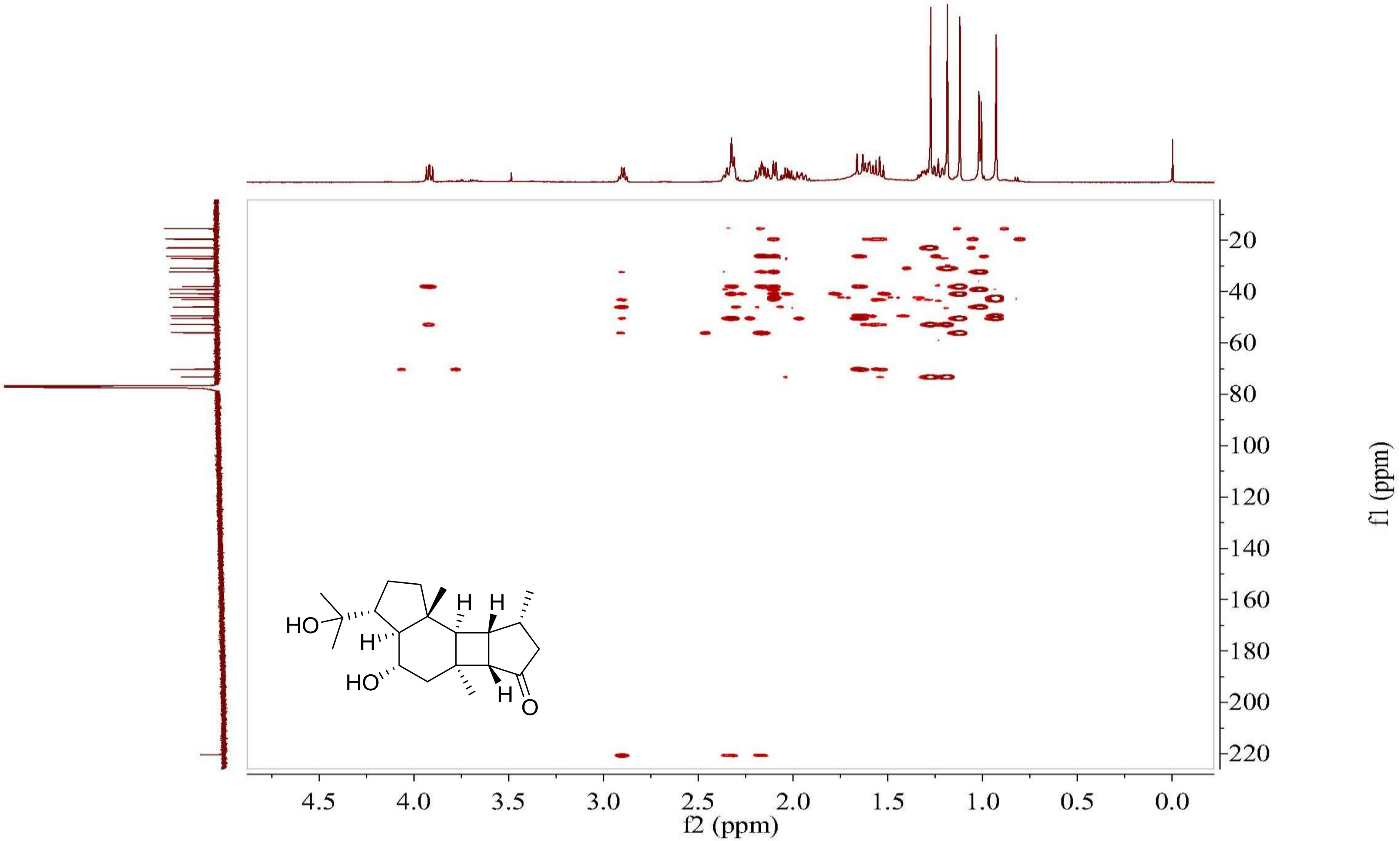

Figure S17. NOESY spectrum of hipposponlachnin B (**2**) in $CDCl_3$

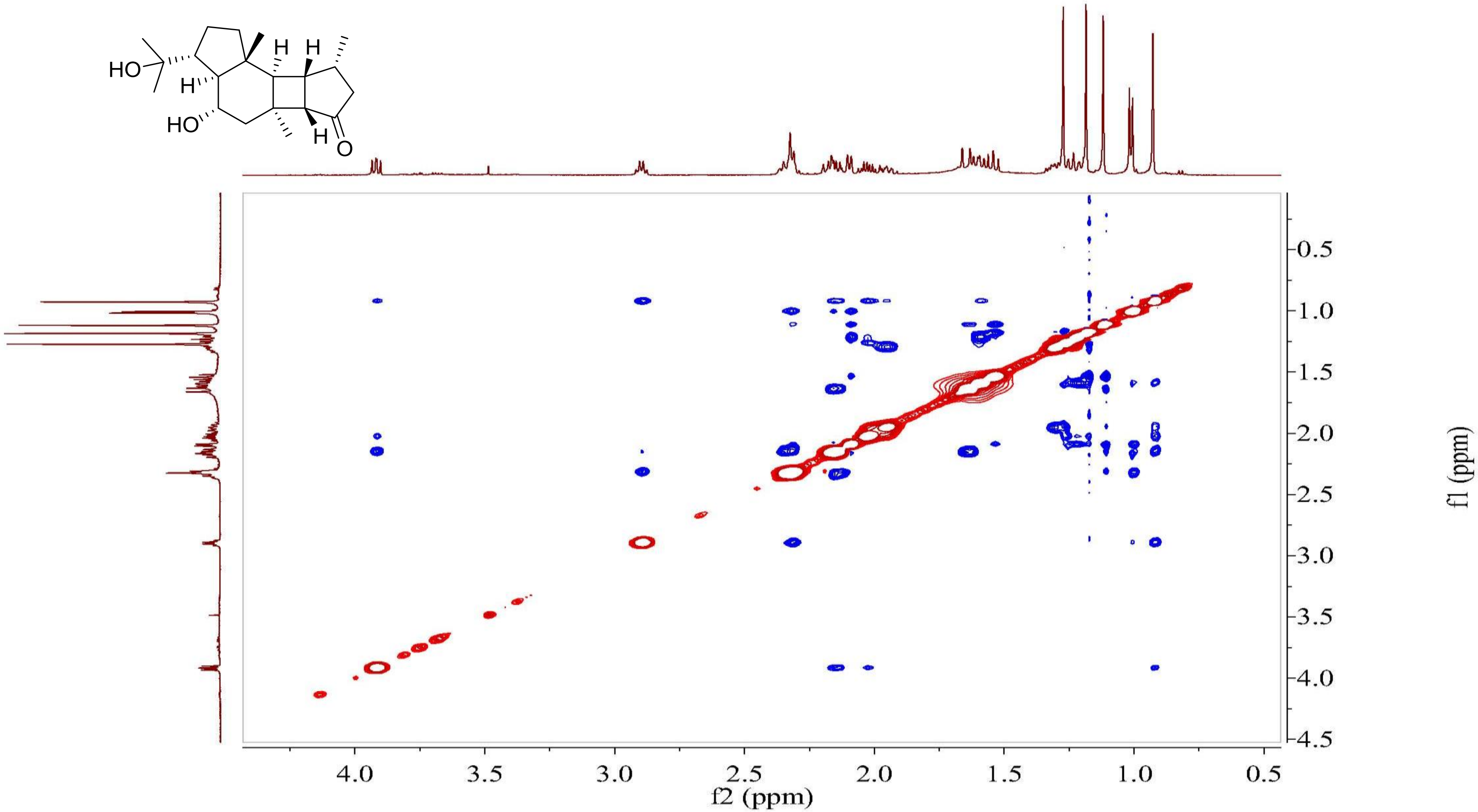

Figure S18. HRESIMS of hipposponlachnin B (**2**)

Tolerance = 10.0 PPM / DBE: min = -1.5, max = 50.0
Selected filters: None

Monoisotopic Mass, Even Electron Ions
8 formula(e) evaluated with 1 results within limits (up to 50 closest results for each mass)
Elements Used:
C: 10-22 H: 10-40 O: 1-4 Na: 1-1

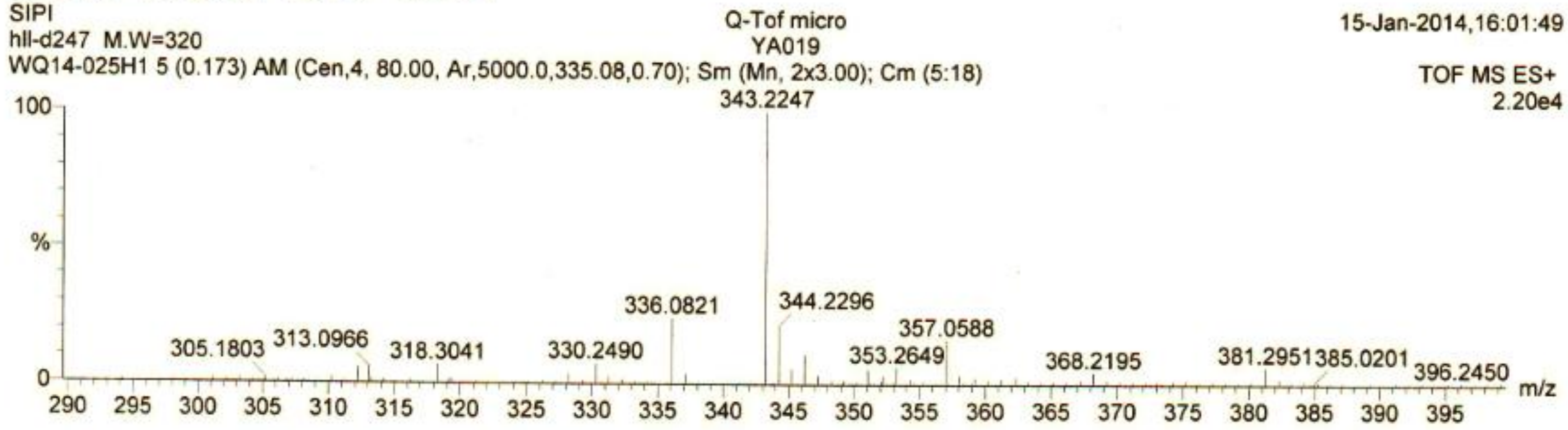

Minimum:	62.00				-1.5		
Maximum:	100.00		5.0	10.0	50.0		
Mass	RA	Calc. Mass	mDa	PPM	DBE	i-FIT	Formula
343.2247	100.00	343.2249	-0.2	-0.6	4.5	130.2	C20 H32 O3 Na

Figure S19. UV spectrum of hipposponlachnin B (**2**) in MeOH

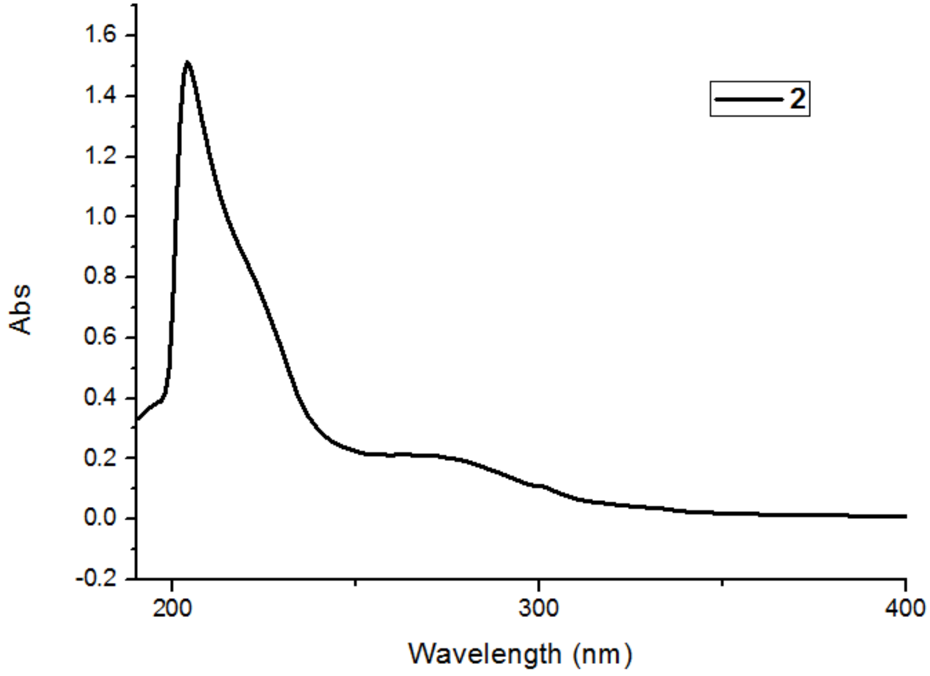

Figure S20. IR spectrum of hipposponlachnin B (**2**)

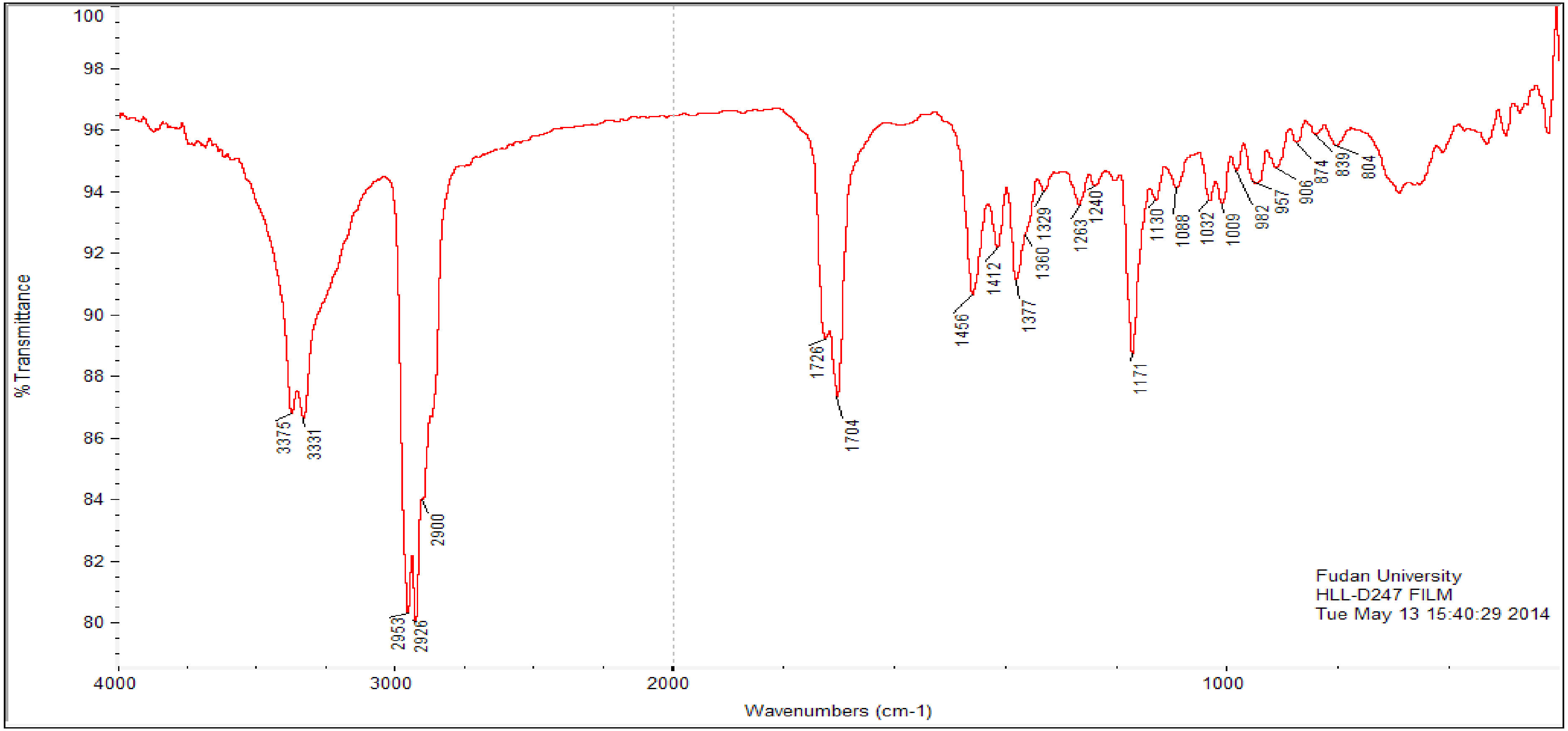

Figure S21. CD spectra of hipposponlachnin A **(1)** and B (**2**) in MeCN.

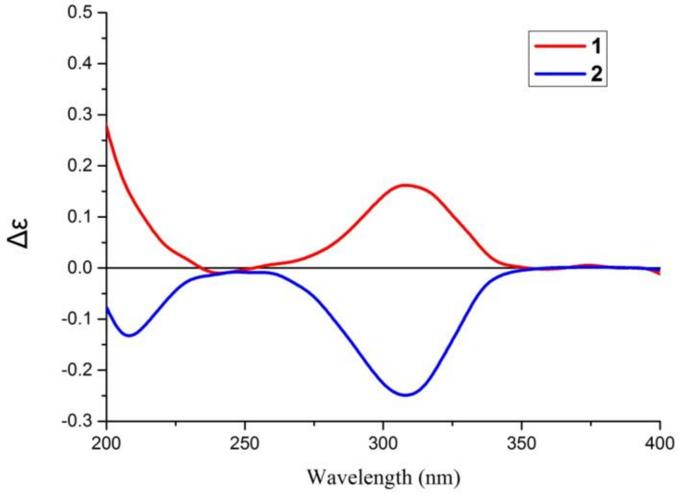

Figure S22. ESIMS of **3**.

Single Mass Analysis
Tolerance = 10.0 mDa / DBE: min = -1.5, max = 50.0
Element prediction: Off
Number of isotope peaks used for i-FIT = 3
Monoisotopic Mass, Even Electron Ions
263 formula(e) evaluated with 7 results within limits (up to 50 best isotopic matches for each mass)
Elements Used:

Mass	Calc. Mass	mDa	PPM	DBE	Formula	i-FIT	i-FIT Norm	Fit Conf %	C	H	O	Na
427.2451	427.2460	-0.9	-2.1	6.5	C24 H36 O5 Na	1756.7	0.207	81.34	24	36	5	1
	427.2436	1.5	3.5	3.5	C22 H37 O5 Na2	1758.3	1.799	16.54	22	37	5	2
	427.2484	-3.3	-7.7	9.5	C26 H35 O5	1760.7	4.212	1.48	26	35	5	
	427.2378	7.3	17.1	12.5	C29 H33 Na2	1762.2	5.727	0.33	29	33		2
	427.2543	-9.2	-21.5	0.5	C19 H39 O10	1762.7	6.218	0.20	19	39	10	
	427.2402	4.9	11.5	15.5	C31 H32 Na	1763.6	7.113	0.08	31	32		1
	427.2426	2.5	5.9	18.5	C33 H31	1764.6	8.128	0.03	33	31		

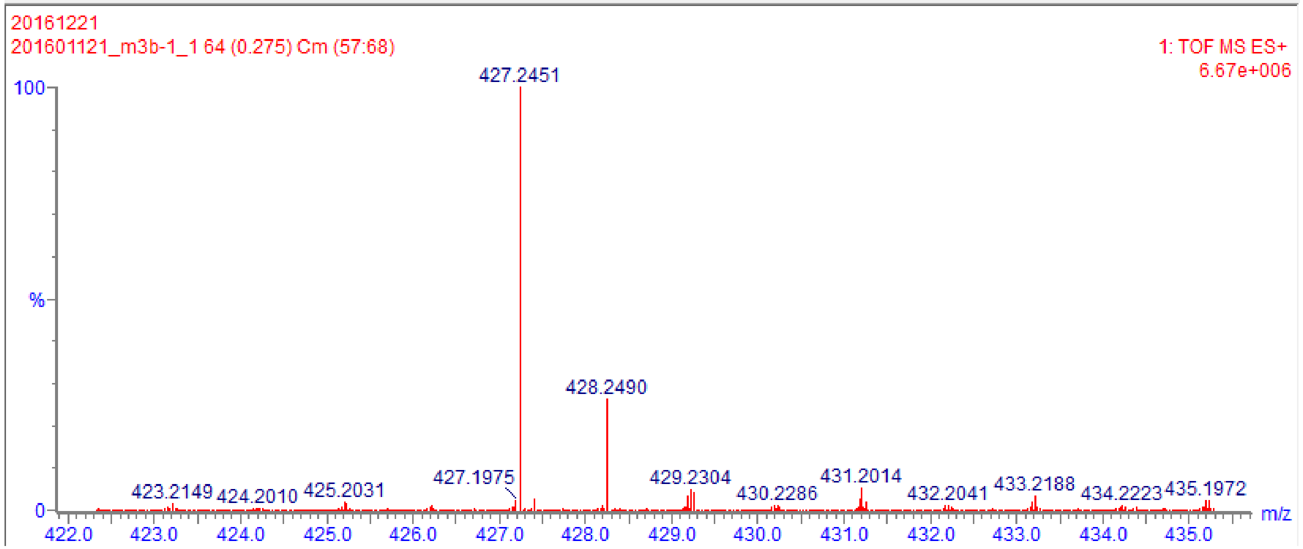

Figure S23. ^{1}H NMR (600 MHz, $CDCl_3$) spectrum of **3**.

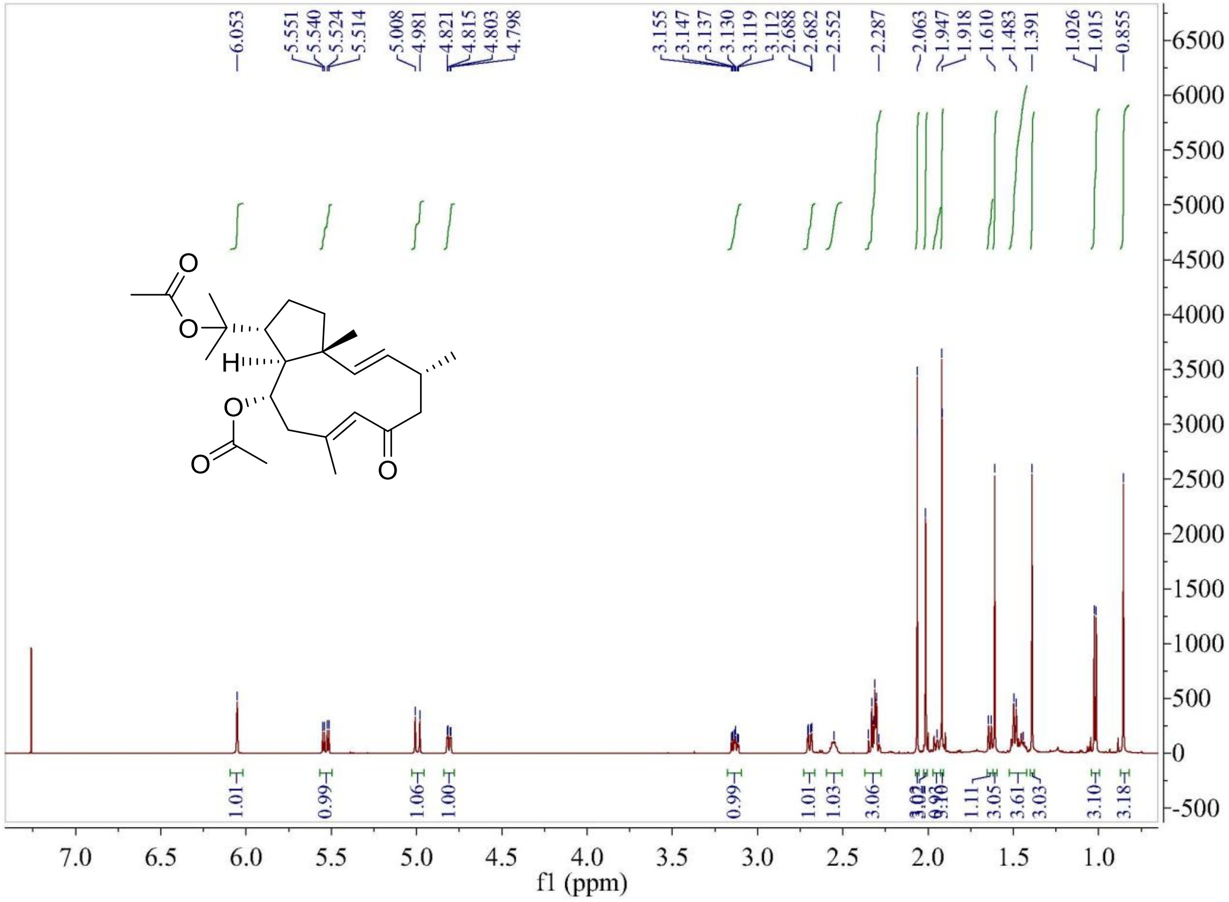

Figure S24. ^{13}C NMR (150 MHz, $CDCl_3$) spectrum of **3**.

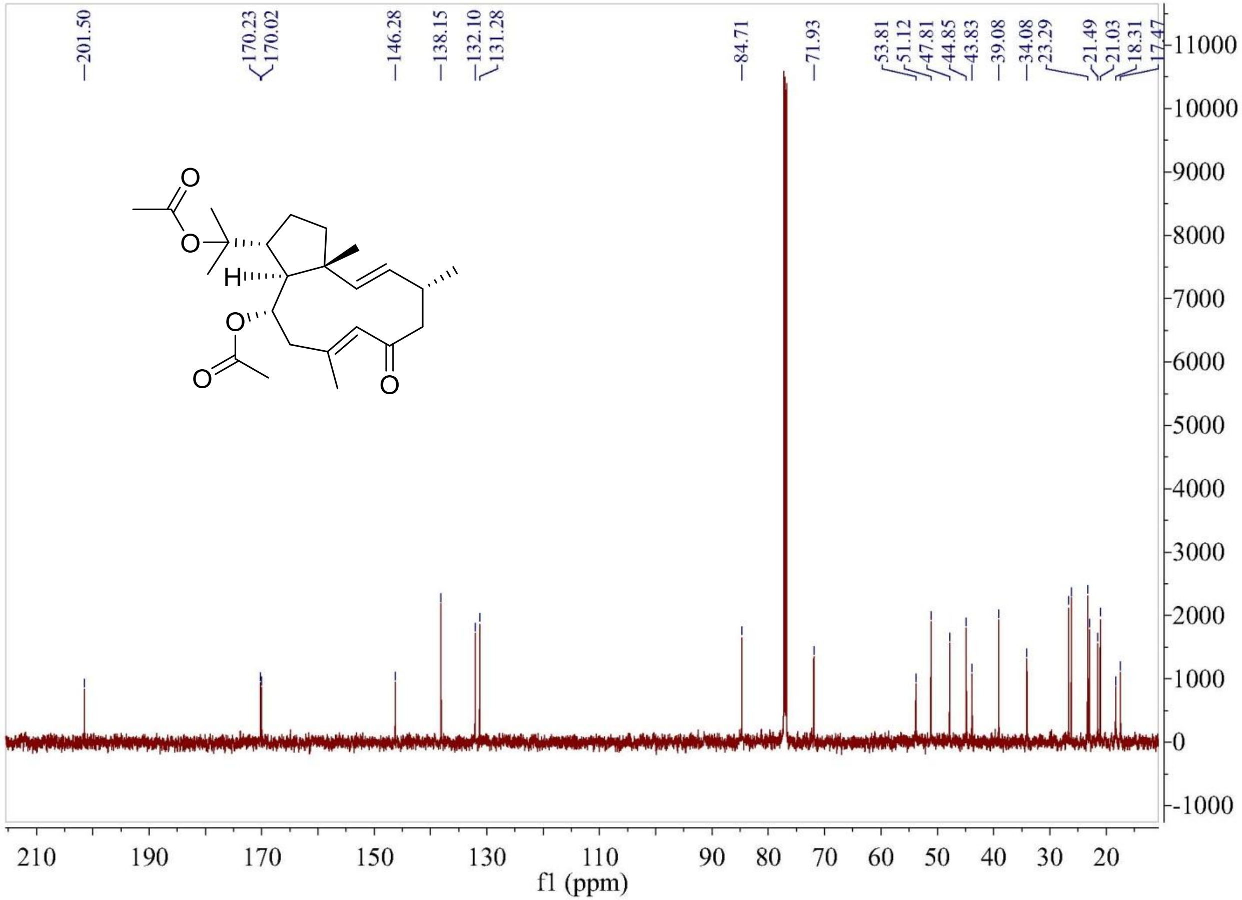

Figure S25. DEPT135 spectrum of **3** in $CDCl_3$.

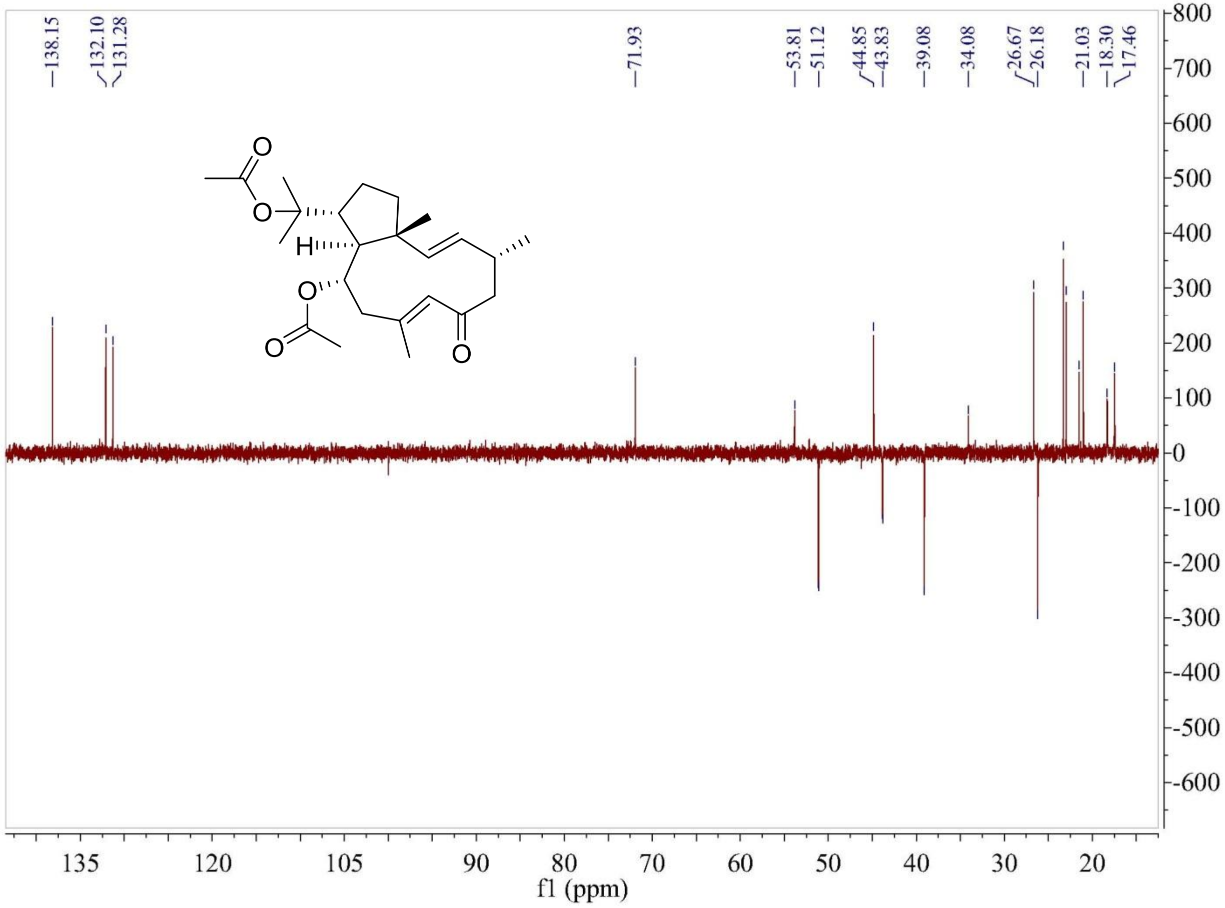

Figure S26. Proposed mechanism of the [2 + 2] cycloaddition of **3** and its 7,8-Z isomer

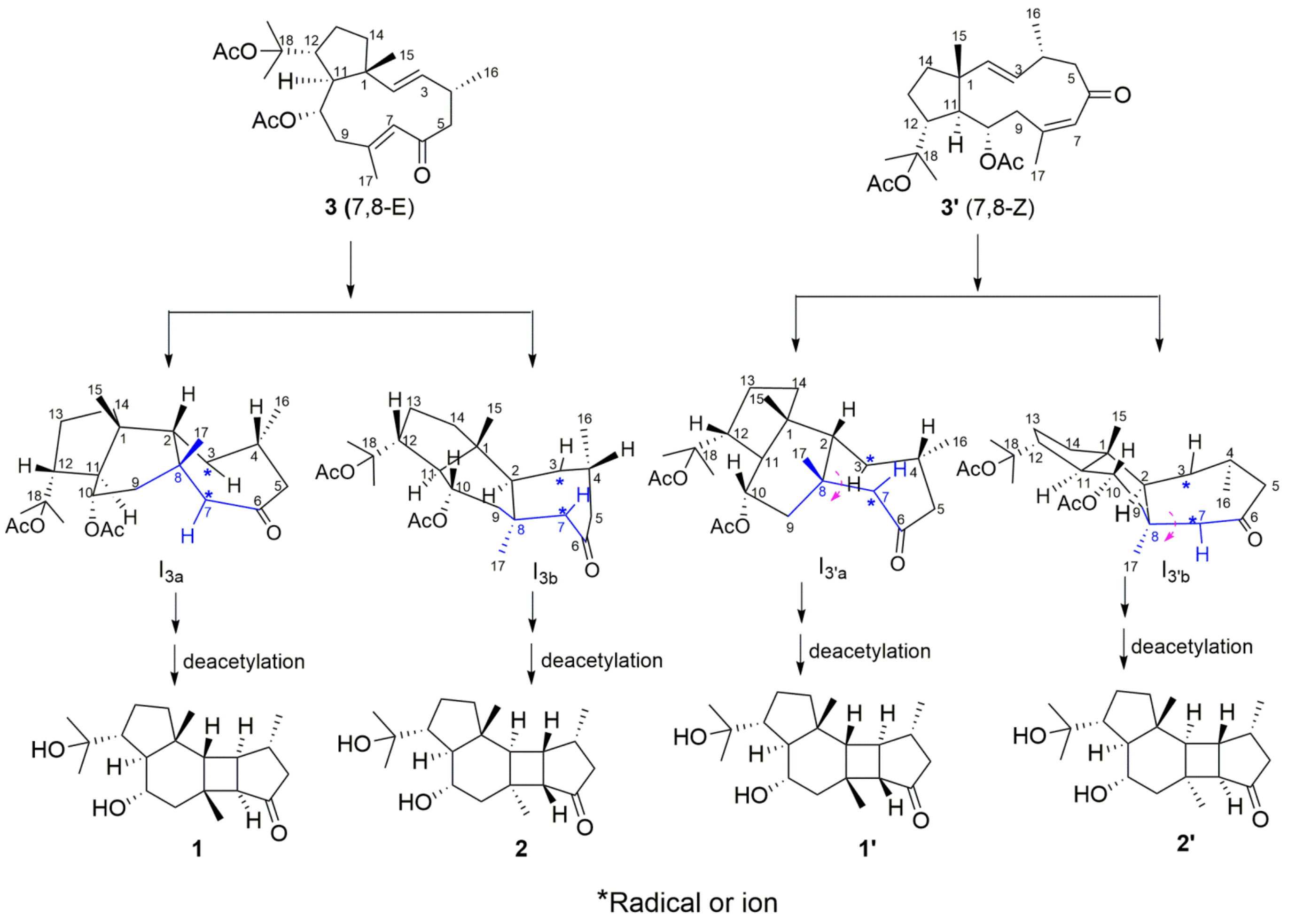

Table S1. Crystal data and structure refinement for hipposponlachnin A (**1**)

Identification code	Cu_dm14258_0m	
Empirical formula	C20 H32 O3	
Formula weight	320.45	
Temperature	140(2) K	
Wavelength	1.54178 Å	
Crystal system	Orthorhombic	
Space group	P 21 21 21	
Unit cell dimensions	a = 6.62210(10) Å	α = 90 °.
	b = 15.2395(3) Å	β = 90 °.
	c = 18.4069(3) Å	γ = 90 °.
Volume	1857.58(6) Å^3	
Z	4	
Density (calculated)	1.146 mg/m^3	
Absorption coefficient	0.588 mm^{-1}	
F(000)	704	
Crystal size	0.200 x 0.120 x 0.050 mm^3	
Theta range for data collection	3.765 to 69.659 °.	
Index ranges	-7<=h<=4, -18<=k<=18, -21<=l<=22	
Reflections collected	8888	
Independent reflections	3177 [R(int) = 0.0311]	
Completeness to theta = 67.679 °	99.4 %	
Absorption correction	Semi-empirical from equivalents	
Max. and min. transmission	0.7532 and 0.6136	
Refinement method	Full-matrix least-squares on F^2	
Data / restraints / parameters	3177 / 0 / 215	
Goodness-of-fit on F^2	1.060	
Final R indices [I>2sigma(I)]	R1 = 0.0354, wR2 = 0.0936	
R indices (all data)	R1 = 0.0377, wR2 = 0.0953	
Absolute structure parameter	0.13(13)	
Extinction coefficient	n/a	
Largest diff. peak and hole	0.131 and -0.188 e.Å^{-3}	

Table S2. Crystal data and structure refinement for hipposponlachnin B (**2**)

Identification code	cu_dm14257_0m	
Empirical formula	C20 H32 O3	
Formula weight	320.45	
Temperature	140(2) K	
Wavelength	1.54178 Å	
Crystal system	Monoclinic	
Space group	P 21	
Unit cell dimensions	a = 8.17720(10) Å	α = 90 °.
	b = 9.71010(10) Å	β = 92.8110(10) °.
	c = 11.2700(2) Å	γ = 90 °.
Volume	893.78(2) $Å^3$	
Z	2	
Density (calculated)	1.191 mg/m^3	
Absorption coefficient	0.611 mm^{-1}	
F(000)	352	
Crystal size	0.200 x 0.100 x 0.020 mm^3	
Theta range for data collection	3.927 to 69.566 °.	
Index ranges	-9<=h<=9, -11<=k<=11, -13<=l<=13	
Reflections collected	6940	
Independent reflections	2953 [R(int) = 0.0248]	
Completeness to theta = 67.679 °	96.3 %	
Absorption correction	Semi-empirical from equivalents	
Max. and min. transmission	0.7532 and 0.6733	
Refinement method	Full-matrix least-squares on F2	
Data / restraints / parameters	2953 / 1 / 215	
Goodness-of-fit on F^2	1.061	
Final R indices [I>2sigma(I)]	R1 = 0.0308, wR2 = 0.0827	
R indices (all data)	R1 = 0.0312, wR2 = 0.0830	
Absolute structure parameter	-0.03(9)	
Extinction coefficient	n/a	
Largest diff. peak and hole	0.188 and -0.143 e.$Å^{-3}$	

CheckCIFPLATON report of hipposponlachnin A (1)

You have not supplied any structure factors. As a result the full set of tests cannot be run.

THIS REPORT IS FOR GUIDANCE ONLY. IF USED AS PART OF A REVIEW PROCEDURE FOR PUBLICATION, IT SHOULD NOT REPLACE THE EXPERTISE OF AN EXPERIENCED CRYSTALLOGRAPHIC REFEREE.

No syntax errors found. CIF dictionary
Please wait while processing .... Interpreting this report

Datablock: cu_dm14258_0m

Bond precision:	C-C = 0.0030 A		Wavelength=1.54178
Cell:	a=6.6221(1)	b=15.2395(3)	c=18.4069(3)
	alpha=90	beta=90	gamma=90
Temperature: 140 K			

	Calculated	Reported
Volume	1857.58(6)	1857.58(6)
Space group	P 21 21 21	P 21 21 21
Hall group	P 2ac 2ab	P 2ac 2ab
Moiety formula	C20 H32 O3	C20 H32 O3
Sum formula	C20 H32 O3	C20 H32 O3
Mr	320.46	320.45
Dx, g cm-3	1.146	1.146
Z	4	4
Mu (mm-1)	0.588	0.588
F000	704.0	704.0
F000'	705.96	
h,k,lmax	8,18,22	7,18,22
Nref	3488[2025]	3177
Tmin,Tmax	0.919,0.971	0.614,0.753
Tmin'	0.889	

Correction method= # Reported T Limits: Tmin=0.614 Tmax=0.753 AbsCorr = MULTI-SCAN

Data completeness= 1.57/0.91 Theta(max)= 69.659

R(reflections)= 0.0354(2970) wR2(reflections)= 0.0953(3177)

S = 1.060 Npar= 215

The following ALERTS were generated. Each ALERT has the format
test-name_ALERT_alert-type_alert-level.
Click on the hyperlinks for more details of the test.

Alert level C

PLAT480_ALERT_4_C Long H...A H-Bond Reported H19B .. O1 .. 2.66 Ang.

Alert level G

PLAT007_ALERT_5_G Number of Unrefined Donor-H Atoms .... 2 Report
PLAT791_ALERT_4_G The Model has Chirality at C1 (Chiral SPGR) R Verify
And 8 other PLAT791 Alerts
More ...

0 **ALERT level A** = Most likely a serious problem - resolve or explain
0 **ALERT level B** = A potentially serious problem, consider carefully
1 **ALERT level C** = Check. Ensure it is not caused by an omission or oversight
10 **ALERT level G** = General information/check it is not something unexpected

0 ALERT type 1 CIF construction/syntax error, inconsistent or missing data
0 ALERT type 2 Indicator that the structure model may be wrong or deficient
0 ALERT type 3 Indicator that the structure quality may be low
10 ALERT type 4 Improvement, methodology, query or suggestion
1 ALERT type 5 Informative message, check

It is advisable to attempt to resolve as many as possible of the alerts in all categories. Often the minor alerts point to easily fixed oversights, errors and omissions in your CIF or refinement strategy, so attention to these fine details can be worthwhile. In order to resolve some of the more serious problems it may be necessary to carry out additional measurements or structure refinements. However, the purpose of your study may justify the reported deviations and the more serious of these should normally be commented upon in the discussion or experimental section of a paper or in the "special_details" fields of the CIF. checkCIF was carefully designed to identify outliers and unusual parameters, but every test has its limitations and alerts that are not important in a particular case may appear. Conversely, the absence of alerts does not guarantee there are no aspects of the results needing attention. It is up to the individual to critically assess their own results and, if necessary, seek expert advice.

Publication of your CIF in IUCr journals

A basic structural check has been run on your CIF. These basic checks will be run on all CIFs submitted for publication in IUCr journals (*Acta Crystallographica*, *Journal of Applied Crystallography*, *Journal of Synchrotron Radiation*); however, if you intend to submit to *Acta Crystallographica Section C* or *E*, you should make sure that full publication checks are run on the final version of your CIF prior to submission.

Publication of your CIF in other journals

Please refer to the *Notes for Authors* of the relevant journal for any special instructions relating to CIF submission.

CheckCIFPLATON report of hipposponlachnin B (**2**)

You have not supplied any structure factors. As a result the full set of tests cannot be run.

THIS REPORT IS FOR GUIDANCE ONLY. IF USED AS PART OF A REVIEW PROCEDURE FOR PUBLICATION, IT SHOULD NOT REPLACE THE EXPERTISE OF AN EXPERIENCED CRYSTALLOGRAPHIC REFEREE.

No syntax errors found.
Please wait while processing ....

CIF dictionary
Interpreting this report

Datablock: cu_dm14257_0m

Bond precision:	C-C = 0.0028 A		Wavelength=1.54178
Cell:	a=8.1772(1)	b=9.7101(1)	c=11.2700(2)
	alpha=90	beta=92.811(1)	gamma=90
Temperature: 140 K			

	Calculated	Reported
Volume	893.78(2)	893.78(2)
Space group	P 21	P 1 21 1
Hall group	P 2yb	P 2yb
Moiety formula	C20 H32 O3	C20 H32 O3
Sum formula	C20 H32 O3	C20 H32 O3
Mr	320.46	320.45
Dx, g cm-3	1.191	1.191
Z	2	2
Mu (mm-1)	0.611	0.611
F000	352.0	352.0
F000'	352.98	
h,k,lmax	9,11,13	9,11,13
Nref	3352[1782]	2953
Tmin,Tmax	0.929,0.988	0.673,0.753
Tmin'	0.885	

Correction method= # Reported T Limits: Tmin=0.673 Tmax=0.753 AbsCorr = MULTI-SCAN

Data completeness= 1.66/0.88 Theta(max)= 69.566

R(reflections)= 0.0307(2906) wR2(reflections)= 0.0818(2953)

S = 1.066 Npar= 215

The following ALERTS were generated. Each ALERT has the format
test-name_ALERT_alert-type_alert-level.
Click on the hyperlinks for more details of the test.

Alert level C

PLAT029_ALERT_3_C _diffrn_measured_fraction_theta_full Low .... 0.963 Note

Alert level G

PLAT007_ALERT_5_G Number of Unrefined Donor-H Atoms .... 2 Report
PLAT791_ALERT_4_G The Model has Chirality at C1 (Chiral SPGR) R Verify
And 8 other PLAT791 Alerts
More ...

0 **ALERT level A** = Most likely a serious problem - resolve or explain
0 **ALERT level B** = A potentially serious problem, consider carefully
1 **ALERT level C** = Check. Ensure it is not caused by an omission or oversight
10 **ALERT level G** = General information/check it is not something unexpected

0 ALERT type 1 CIF construction/syntax error, inconsistent or missing data
0 ALERT type 2 Indicator that the structure model may be wrong or deficient
1 ALERT type 3 Indicator that the structure quality may be low
9 ALERT type 4 Improvement, methodology, query or suggestion
1 ALERT type 5 Informative message, check

It is advisable to attempt to resolve as many as possible of the alerts in all categories. Often the minor alerts point to easily fixed oversights, errors and omissions in your CIF or refinement strategy, so attention to these fine details can be worthwhile. In order to resolve some of the more serious problems it may be necessary to carry out additional measurements or structure refinements. However, the purpose of your study may justify the reported deviations and the more serious of these should normally be commented upon in the discussion or experimental section of a paper or in the "special_details" fields of the CIF. checkCIF was carefully designed to identify outliers and unusual parameters, but every test has its limitations and alerts that are not important in a particular case may appear. Conversely, the absence of alerts does not guarantee there are no aspects of the results needing attention. It is up to the individual to critically assess their own results and, if necessary, seek expert advice.

Publication of your CIF in IUCr journals

A basic structural check has been run on your CIF. These basic checks will be run on all CIFs submitted for publication in IUCr journals (*Acta Crystallographica*, *Journal of Applied Crystallography*, *Journal of Synchrotron Radiation*); however, if you intend to submit to *Acta Crystallographica Section C* or *E*, you should make sure that full publication checks are run on the final version of your CIF prior to submission.

Publication of your CIF in other journals

Please refer to the *Notes for Authors* of the relevant journal for any special instructions relating to CIF submission.
